# *Medicago truncatula* Phytoglobin 1.1 controls symbiotic nodulation and nitrogen fixation via the regulation of nitric oxide concentration

**DOI:** 10.1111/nph.16462

**Published:** 2020-03-14

**Authors:** Antoine Berger, Sophie Guinand, Alexandre Boscari, Alain Puppo, Renaud Brouquisse

**Affiliations:** ^1^ Institut Sophia Agrobiotech UMR INRAE 1355 CNRS 7254 Université Côte d'Azur 400 route des Chappes, BP 167 06903 Sophia Antipolis France

**Keywords:** legume, *Medicago truncatula*, nitric oxide, nitrogen‐fixing symbiosis, nodule, phytoglobin

## Abstract

- In legumes, phytoglobins (Phytogbs) are known to regulate nitric oxide (NO) during early phase of the nitrogen‐fixing symbiosis and to buffer oxygen in functioning nodules. However, their expression profile and respective role in NO control at each stage of the symbiosis remain little‐known.
- We first surveyed the *Phytogb* genes occurring in *Medicago truncatula* genome. We analyzed their expression pattern and NO production from inoculation with *Sinorhizobium meliloti* up to 8 wk post‐inoculation. Finally, using overexpression and silencing strategy, we addressed the role of the Phytogb1.1‐NO couple in the symbiosis.
- Three peaks of *Phytogb* expression and NO production were detected during the symbiotic process. NO upregulates *Phytogbs1* expression and downregulates *Lbs* and *Phytogbs3* ones. *Phytogb1.1* silencing and overexpression experiments reveal that Phytogb1.1‐NO couple controls the progression of the symbiosis: high NO concentration promotes defense responses and nodular organogenesis, whereas low NO promotes the infection process and nodular development. Both NO excess and deficiency provoke a 30% inhibition of nodule establishment. In mature nodules, Phytogb1.1 regulates NO to limit its toxic effects while allowing the functioning of Phytogb‐NO respiration to maintain the energetic state.
- This work highlights the regulatory role played by Phytogb1.1–NO couple in the successive stages of symbiosis.

## Abstract 2

See also the Commentary on this article by Singh *et al*., **227**: 5–7.

## Introduction

The symbiotic interaction between legumes and Rhizobium bacteria results in the formation of a new root organ, the nodule, whose main function is the reduction and fixation of atmospheric nitrogen (N_2_). The process starts with the mutual recognition of both the plant and the bacterial partners. Bacteria enter the root hairs via a specific structure, the infection thread, while some cells of the root cortex divide to form the nodule (Long, 2001). Inside the infection thread that progresses and reaches the cortical cells, bacteria divide and are released into the host cells of the developing nodule. Bacteria then differentiate into bacteroids that reduce N_2_ via nitrogenase activity (Oldroyd & Downie, 2008). Indeterminate nodules such as those of alfalfa, clover or pea possess a persistent meristem and comprise four distinct zones: zone I, the meristematic cells; zone II, where the bacteria enter the host cells and differentiate into bacteroids; zone III, where bacteroids reduce N_2_ to ammonia (NH_3_); and zone IV, characterized by the breakdown of the symbiosis and the onset of senescence (Timmers *et al.*, 2000). As nitrogenase is irreversibly inhibited by traces of oxygen (O_2_), N_2_ fixation requires the microaerophilic conditions found in nodules (Appleby, 1992).

Nitric oxide (NO) is a bioreactive gaseous molecule found in living organisms. In plants, it participates in the regulation of developmental stages, from germination to senescence (Bruand & Meilhoc, 2019; González‐Gordo *et al*., 2019; Stasolla *et al.*, 2019), and in the response to many abiotic stresses, including hypoxia (Simontacchi *et al.*, 2015). NO is produced during symbiotic interactions, and many studies report its presence during legume–rhizobia symbiosis. NO production is transiently induced in the roots of *Lotus japonicus* and *Medicago sativa* a few hours post‐inoculation (hpi) with their bacterial partners (Nagata *et al.*, 2008; Fukudome *et al.*, 2016). NO is also produced in shepherd's crooks of root hairs, infection threads, and nodule primordia (del Giudice *et al.*, 2011). In mature nodules, NO was found complexed with leghemoglobin (Lb) (Maskall *et al.*, 1977; Mathieu *et al.*, 1998; Sánchez *et al.*, 2010) and its presence was mainly associated with the N_2_‐fixing zone (Baudouin *et al.*, 2006). Cam *et al. *(2012) observed that NO is also produced between the N_2_‐fixing and senescence zones at the end of the symbiotic process. Considered together, these observations mean that NO is present at various time‐points of the symbiotic process (Hichri *et al.*, 2015, 2016; Meilhoc *et al*., 2015) and the question is raised as to its physiological roles in different times and spaces of symbiotic interaction (Hichri *et al.*, 2015; Berger *et al.*, 2019).

The toxic, signaling or metabolic roles of NO depend on its concentration at the action site (Mur *et al.*, 2013). Therefore, its concentration must be tightly controlled. Several NO sources have been identified in plants, including reductive and oxidative pathways (Mur *et al.*, 2013). The turnover of NO metabolism and messaging depends on the activity of S‐nitrosoglutathione reductase that controls the S‐nitrosoglutathione pool, a major reservoir of NO (Leterrier *et al.*, 2011; Yun *et al.*, 2016; Astier *et al.*, 2018). NO removal was mainly ascribed to hemoglobins (Hbs) (Gupta *et al.*, 2011). Plant Hbs, renamed phytoglobins (Phytogbs; Hill *et al*., 2016), have been classified into six categories, including: Phytogb0 – nonsymbiotic hemoglobin (nsHb); Phytogb1 – class 1 nonsymbiotic hemoglobin (nsHb‐1); Phytogb2 – class 2 nonsymbiotic hemoglobin (nsHb‐2); SymPhytogb – symbiotic hemoglobin (symHb); Lb – leghemoglobin (Lb); and Phytogb3 – class 3 truncated hemoglobin (trHb) (Hill *et al*., 2016). Three types of Hbs were described in legumes and are expressed during N_2_‐fixing symbiosis: Phytogb1, Lb and Phytogb3 (Bustos‐Sanmamed *et al.*, 2011). Owing to their very high affinity for O_2_ and NO, Phytogb1 are capable of recovering traces of O_2_ and NO to convert them to nitrate at very low O_2_ concentrations (Gupta *et al.*, 2011; Igamberdiev *et al.*, 2011). Phytogb1 scavenge NO and, in return, NO functions as an inducer of Phytogb1 (Nagata *et al.*, 2009; Hill, 2012). Thus, the ‘Phytogb1–NO’ couple forms a feedback loop allowing a rapid NO concentration regulation. Such a regulation was shown to occur during early steps of N_2_‐fixing (Nagata *et al.*, 2009; Murakami *et al*., 2011) and mycorrhizal (Martinez‐Medina *et al.*, 2019) symbiosis. In *L. japonicus* nodules, the overexpression of *LjPhytogb1* reduces NO content and enhances N_2_ fixation (Shimoda *et al.*, 2009; Fukudome *et al*., 2018), suggesting that reversible inhibition of nitrogenase is relieved by the scavenging of NO by Phytogb1. Functional nodules are characterized by a microoxic environment. In many root systems under microoxic conditions, NO increases and is scavenged by Phytogb1 to generate ATP in a Phytogb–NO respiratory cycle (Igamberdiev & Hill, 2004). This cycle contributes to the preservation of NAD(P)H/NAD(P)^+^ and ATP : ADP ratios in hypoxic cells and keeps their viability (Igamberdiev *et al.*, 2010). Accumulating data support the existence of a Phytogb–NO cycle in legume nodules: a strong increase of LbNO complex formation is observed in nodules of soybean plants submitted to hypoxia (Meakin *et al.*, 2007; Sanchez *et al*., 2010), and the inhibition of the Phytogb–NO cycle strongly decreases the ATP : ADP ratio in *M. truncatula* nodules (Horchani *et al.*, 2011). Lbs accumulate at a millimolar concentration in the cytoplasm of infected nodular cells (Appleby, 1992). They are considered as markers of N_2_‐fixing symbiosis and their protein abundance correlates with the N_2_‐fixation activity of the nodules. Lbs buffer free O_2_ in the nanomolar range, thus avoiding the inactivation of nitrogenase while maintaining a high flux of O_2_ for respiration (Appleby, 1992). It has been shown that deoxy‐Lb binds to NO with high affinity to form stable complexes in soybean and that Lb could act as a NO and peroxynitrite scavenger (Herold & Puppo, 2005). Phytogb3 are induced in *M. truncatula* (Vieweg *et al.*, 2005) and *Frankia* (Niemann & Tisa, 2008; Coats *et al.*, 2009) N_2_‐fixing symbiosis and have been proposed to be involved in NO scavenging.

Although analyzed at specific time‐points of the N_2_‐fixing symbiosis, neither *Phytogbs* expression nor NO production have been investigated in respect of the entire symbiotic process. In this work, we first survey *Phytogb* genes in the *M. truncatula* genome. Then, we analyze Phytogbs expression and NO production from the first hours of symbiotic interaction up to 8 wk post‐inoculation (wpi), when the interaction breaks down. Using overexpression and silencing strategy, we investigate further the role of Phytogb1.1 in the regulation of NO concentration during the first days of symbiosis establishment and in N_2_‐fixing nodules. Based on our data, we discuss the roles of Phytogb1.1 and NO during the different stages of symbiosis.

## Materials and Methods

### Plants growth and inoculation conditions

*Medicago truncatula* (cv Jemalong A17) were scarified, sterilized and germinated as in del Giudice *et al. *(2011). Seedlings were cultivated and inoculated with *Sinorhizobium meliloti* 2011 strain either in Petri dishes as in del Giudice *et al*. (2011), or in planters as in Horchani *et al. *(2011). A basic intake of 0.2 mM KNO_3_ is provided to crops on Petri dishes and planters. Cultures in Petri dishes were used for short‐term experiments up to 14 d post‐inoculation (dpi), while those in planters were used for long‐term experiments up to 8 wpi. Roots and/or nodules were harvested at various times of the kinetics. For short‐term experiments, 2‐cm‐long root segments corresponding to the infection zone (del Giudice *et al.*, 2011) were harvested for gene expression and NO production; for long‐term experiments only nodules were used.

### Plasmid constructions

For overexpression constructions, the complete cDNA of *M. truncatula Phytogb1.1* was amplified by PCR and cloned in pDONR207 vector. This sequence was introduced either in the pK7WG2D vector under the control of the 35S promoter (named 35s::*Phytogb1.1*) by simple Gateway reaction or in pKm43GWrolDGFP by multiple Gateway reaction according to the manufacturer's instructions (Invitrogen). For the multiple Gateway reaction, *Phytogb1.1* open reading frams was placed under the control of the NCR001 gene promoter (Mergaert *et al.*, 2003) (named NCR::*Phytogb1.1*). For RNAi constructions, a common region of *c.* 200 bp found in the complete cDNA of *M. truncatula Phytogb1.1* was amplified by PCR using the couple primers RNAi–Phytogb1.1. This sequence was introduced into either the pK7GWIW2D vector (Karimi *et al.*, 2002) (named RNAi*::Phytogb1.1*) or the pK7GWIWG5D(II) vector (Horchani *et al.*, 2011) (named NCR‐RNAi::*Phytogb1.1*). Primer sequences are provided in Supporting Information Table S1.

### Roots transformation by *Agrobacterium* rhizogenes

The different constructions were introduced into *A. rhizogenes* strain *Arqua1* (Quandt & Hynes, 1993). *Medicago truncatula* plants were transformed with *A. rhizogenes* according to Boisson‐Dernier *et al.* (2001). Control plants were transformed with *A. rhizogenes* containing either the pK7GWIGW2D or the pK7WG2D empty vectors. Transgenic roots were selected under a Leica MZ FLIII fluorescence stereomicroscope (Leica, Wetzlar, Germany) based on the green fluorescence protein signal at 2 wk after germination. After the removal of nontransgenic roots, composite plants were transferred to new plates containing Fahräeus medium supplemented with 0.2 mM NH_4_NO_3_ and without antibiotic. For the construction under the control of NCR001 promoter, *M. truncatula* plants were transformed with *A. rhizogenes* according to Vieweg *et al.* (2005). After the selection of transformed hairy roots based on the fluorescent marker at 21 d after transformation, composite plants harboring transgenic roots were placed in planters and inoculated 3 d later with *S. meliloti* strain.

### Measurement of NO production

Nitric oxide detection was performed as in Horchani *et al. *(2011) using the 4,5‐diaminofluorescein probe (DAF‐2; Sigma‐Aldrich) with the following changes. Either nodules (20–30 mg FW) or root segments (50–100 mg FW) were incubated in 1 ml of detection buffer (10 mM Tris‐HCl pH 7.4, 10 mM KCl) in the presence of 10 μM DAF‐2. As a control, NO production was measured in the same experimental system through the use of the Cu(II) fluorescein (CuFL) fluorescent probe (Strem Chemicals, Bischheim, France) instead of DAF‐2 in the detection buffer as described in Horchani *et al. *(2011). Similar results were obtained with both probes. The production of NO was measured with a spectrofluorimeter‐luminometer (Xenius, Safas, Monaco).

### RNA isolation, reverse transcription and genes expressions

RNAs were isolated from 100 mg of frozen material ground in liquid N_2_ using the RNAzol following the manufacturer's recommendations (Sigma‐Aldrich). RNA quality was checked and DNase treatment was carried out before the synthesis by GoScript reverse transcriptase (Promega) of the cDNAs. The quantitative reverse transcription polymerase chain reaction (RT‐qPCR) was done with the Go‐Taq qPCR master Mix kit according to the manufacturer's instructions (Promega). RT‐qPCR data analyses were carried out using rqpcrbase, an R package working in the R computing environment for analysis of quantitative real‐time PCR data (Hilliou & Tran, 2013). The expression of the different genes was normalized against two housekeeping genes, *Mtc27* (Van de Velde *et al.*, 2006) and *Mta38* (del Giudice *et al.*, 2011). RT‐qPCR analyses were carried out in triplicate, using the primers reported in Table S1.

### NO donor treatments

Plants were treated with 0.5 mM of either diethylamine‐NONOate (DEA‐NO) or DEA solutions. Two hundred microliters of solution were added along the whole length of the roots at 2 h before inoculation with *S. meliloti* and then every 24 h for 4 d. Control plants were treated with water. After 4 d, plants were either analyzed for gene expression or transferred to a NO donor free medium and grown for an 10 additional days before measurement of nodule number.

### Nitrogen‐fixing capacity measurement

Nitrogenase activity of nodules was determined *in vivo* by measuring acetylene reducing activity (ARA; Hardy *et al.*, 1968). Nodulated roots were harvested and incubated at 30°C for 1 h in rubber‐capped tubes containing a 10% acetylene atmosphere. Ethylene concentrations were determined by GC (Agilent GC 6890N; Agilent Technologies, Les Ulis, France) equipped with a GS‐Alumina (Agilent Technologies) separating capillary column. Three independent biological replicates have been performed with five technical replicates per biological assay.

### Extraction and measurement of nodule adenine nucleotides

Adenine nucleotides were extracted and measured as in Horchani *et al. *(2011). Adenine nucleotides were measured in a Xenius spectrofluorimeter‐luminometer using the ATPlite one‐step assay system (Perkin‐Elmer, Villebon‐sur‐Yvette, France) according to the manufacturer's instructions.

### Phylogeny

The phylogeny data were obtained using the one‐click mode of the website (http://www.phylogeny.fr; Dereeper *et al.*, 2008) which includes a sequence alignment using the muscle and gblocks programs. Phylogenetic reconstruction was done with the phyml program using the maximum likelihood method. Nodes with a robustness < 80% were pooled in the same phylogenetic subgroup.

## Results

### *Medicago truncatula* phytoglobin family

Research in genomic and protein libraries (JCVI, https://www.jcvi.org/medicago‐truncatula‐genome‐database; NCBI, https://www.ncbi.nlm.nih.gov/; UniProt, https://www.uniprot.org/) revealed that the *M. truncatula* genome contains 17 Phytogb genes. Phylogenetic analysis of protein sequences of *M. truncatula* Phytogbs, compared with *Glycine max*, *L. japonicus* and *Arabidopsis thaliana* Phytogbs, confirmed the presence of three Phytogb classes (Fig. S1). Three Phytogb1, two Phytogb3 and 12 Lbs were identified in *M. truncatula*, whereas *G. max* and *L. japonicus* possess two Phytogb1, and only four and six Lbs, respectively (Fig. S1). This large number of Lbs with distinct protein sequences (Fig. S2) and Affymetrix expression patterns (Fig. S3) highlights the still unresolved but different roles and locations of each of them within the *M. truncatula* nodule. *Medicago truncatula Lbs* genes are found in chromosomes 1, 5 and 7 (Fig. S4a). Five *Lbs* genes are located close to each other in a 265 kb region of chromosome 5. This cluster of genes could be the origin of gene duplication events and explain the large number of *Lbs* in *M. truncatula* (Storz, 2016). *Phytogb1.1* and *Phytogb1.2* are located in a restricted area in chromosome 4 (no information is available on the chromosomal location of *Phytogb1.3*). The two *Phytogb3* genes previously identified by Vieweg *et al. *(2005) are located in the chromosomes 1 and 3. The ‘exon‐intron’ structure analysis shows that most of the *Phytogbs* contain four exons and three introns (Fig. S4b). This structure, already described in the *Phytogbs* of *L. japonicus* (Bustos‐sanmamed *et al.*, 2011), is representative of the ancestral hemoglobin gene (Hardison, 1998). The meme analysis tool (http://meme‐suite.org) was used to identify conserved motifs in the protein sequences of *M. truncatula* Phytogbs (Fig. S5). This analysis identified four highly conserved motifs, one of which is involved in heme binding and another responsible for NO dioxygenase activity (Fig. S5) (Smagghe *et al*., 2008). The protein sequence of Phytogb1.1 is similar to that of Lbs, except for Lb8 and Lb11. Phytogb1.2 and 1.3 have a sequence twice as long and a repetition of the four protein motifs (Figs S4b, S5) that correspond to the same repetitions of exons in the gene sequence. This doubling of the gene and protein sequence is not observed in Phytogbs of *A. thaliana* or legumes such as *G. max*, *L. japonicus* and *P. sativum*, but it is found in *Trifolium subterraneum* and *Vicia faba* (http://www.coolseasonfoodlegume.org/). Interestingly, the two *Phytogb3* genes have only the heme binding domain (Fig. S5), which raises the question of whether they possess NO dioxygenase activity.

Considering the confusion in the name of the *M. truncatula Phytogb* genes in the literature, and based on Mt4.0 database classification, we propose to homogenize their nomenclature. Nomenclature, Affymetrix, gene (Mt4.0 genome version from Noble database), and Symbimics accession codes (Roux *et al.*, 2014) of the 17 *MtPhytogb* genes are listed in Table 1.

**Table 1:**
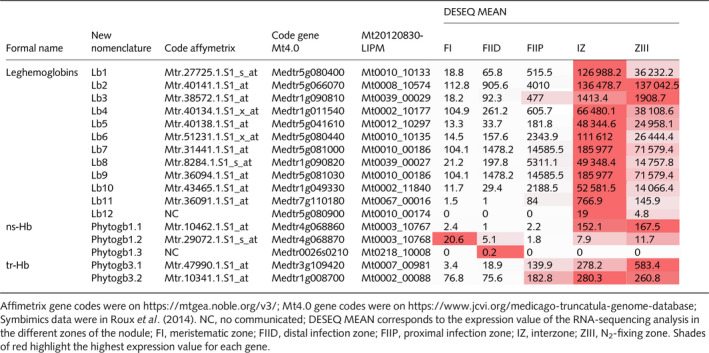
Nomenclature, access codes and Symbimics expression of *Medicago truncatula* phytoglobins.

### Phytoglobin genes expression during the symbiotic process

**Fig. 1:**
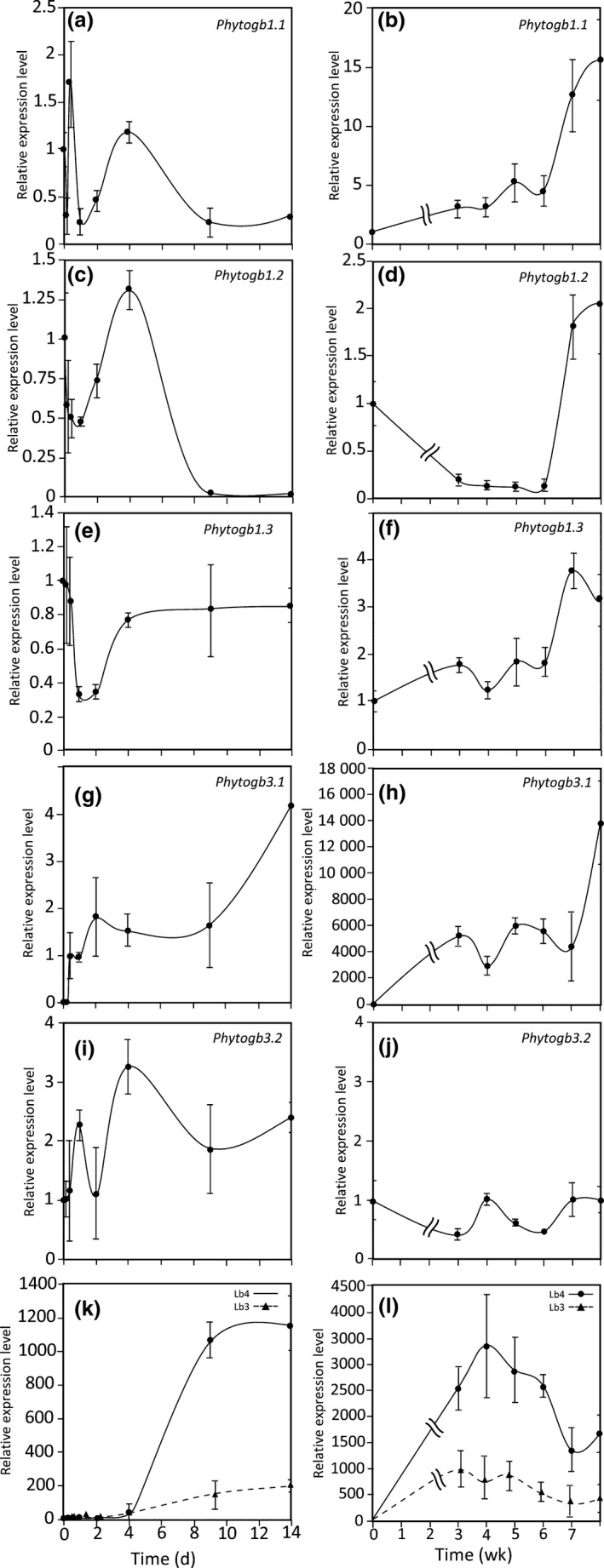
Expression of *Medicago truncatula Phytogb1*, *Phytogb3* and *Lb* genes during the symbiotic process: short‐term kinetic 14 d post‐inoculation (dpi) (a, c, e, g, i, k); long‐term kinetic 8 wk post‐inoculation (wpi) (b, d, f, h, j, l). Expression of *Phytogb1.1* (a, b), *Phytogb1.2* (c, d), *Phytogb1.3* (e, f), *Phytogb3.1* (g, h), *Phytogb3.2* (i, j), and *Lb3* and *4* (k, l). Data are means ± SE (*n* = 3). Each measurement was done in triplicate.

*Medicago truncatula Phytogb* expression patterns were analyzed from 0 to 8 wpi. Two types of *M. truncatula* cultures were used: a short‐term culture from 0 to 14 dpi, and a long‐term culture up to 8 wpi. As compared with its expression level in noninoculated roots, *Phytogb1.1* expression exhibited first a 75% drop at 4 hpi with *S. meliloti*, and two transient peaks at 10 hpi and 4 dpi (Fig. 1a). It then increased progressively up to 5 wpi and strongly at 7–8 wpi, at the onset of nodule senescence (Fig. 1b). After a 50% decrease during the first hours of the interaction, *Phytogb1.2* expression transiently peaked at 4 dpi and strongly increased at 7–8 wpi in senescent nodules (Fig. 1c,d). After a transient decrease at 1–2 dpi, the expression of *Phytogb1.3* changed only slightly up to 6 wpi and then peaked at 7 wpi when senescence is initiated (Fig. 1e,f). *Phytogb3.1* expression, undetectable in noninoculated roots, was rapidly induced at 10 hpi. Its expression remained steady up to 9 dpi, then increased to reach a plateau between 3 and 7 wpi, and finally increased strongly at 8 wpi (Fig. 1g,h). Except for a peak at 4 dpi, the expression of *Phytogb3.2* fluctuated only moderately and remained stable during the whole symbiotic process (Fig. 1i,j).

The analysis of Affymetrix and Symbimics data (Fig. S3; Table S1) showed that the 12 *Lb* genes exhibit a similar expression pattern and are expressed in the nodule interzone II–III and zone III. Therefore, to avoid analyzing the expression of the 12 *Lb* genes, we used *Lb4* and *Lb3*, whose expression is average among the different *Lb*, as representative *Lb* markers (Fig. S3). Their expression remained close to the detection limit up to 4 dpi (Fig. 1k). Then, it strongly increased to reach a maximum between 3 and 5 wpi, when the N_2_‐fixing activity of nodules is maximal, and finally decreased when the nodules enter in senescence between 6 and 8 wpi (Fig. 1l).

**Fig. 2:**
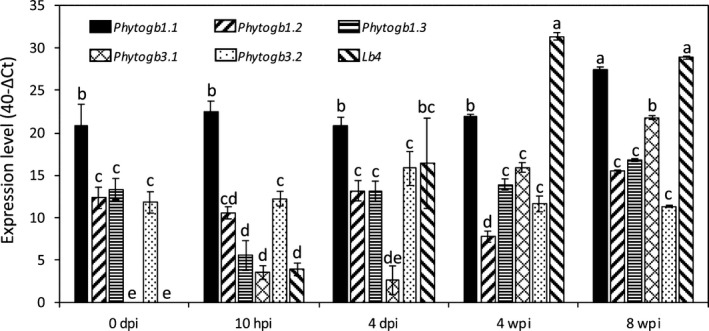
Expression of *Medicago truncatula Phytogb* genes at various times of the symbiotic process. The reference value ‘1’ was attributed to the first time when the cycle threshold (*Ct*) of the analyzed gene was significantly detectable. Comparative expression levels between genes are given on a logarithmic scale expressed as 40 − Δ*Ct*, where Δ*Ct* is the difference in quantitative reverse transcription polymerase chain reaction threshold cycle number between the respective gene and the reference gene; the number 40 was chosen because the PCR run stops after 40 cycles (Bari *et al.*, 2006; Truong *et al.*, 2015). Data are means ± SE (*n* = 3). Values followed by different letters are significantly different according to one‐way ANOVA analysis followed by a Fisher test (*P* < 0.05). dpi, d post‐inoculation; hpi, h post‐inoculation; wpi, wk post‐inoculation.

The expression levels of *Phytogb* as compared with each other, before inoculation and at four time‐points in the symbiosis, are reported in Fig. 2. Several features emerged from this analysis: predictably, *Lb4* was more highly expressed than *Phytogb1* and *Phytogb3* in 4 wpi N_2_‐fixing nodules; whereas *Phytogb1.1*, *1.2*, *1.3* and *3.2* were constitutively expressed in roots and nodules, *Phytogb3.1* became one of the most highly expressed in mature nodules, suggesting a particular role in N_2_ fixation; with the exception of *Phytogb3.2*, all the *Phytogbs* analyzed in this study were highly expressed in the senescent nodules at 8 wpi; in noninoculated roots, *Phytogb1.1* was the most strongly expressed *Phytogb* and it remained highly expressed throughout the symbiotic process.

### NO production during the symbiotic process

**Fig. 3:**
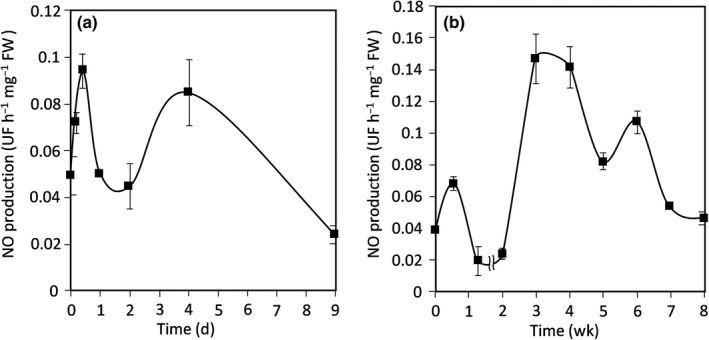
Nitric oxide (NO) concentration during the *Medicago truncatula* symbiotic process: (a) short‐term kinetic 14 d post‐inoculation (dpi); (b) long‐term kinetic 8 wk post‐inoculation (wpi). The fluorescence intensity of the NO production was measured using the 4,5‐diaminofluorescein probe (DAF‐2; Sigma‐Aldrich). Data are means ± SE (*n* = 3). Each measurement was done in triplicate.

Nitric oxide production was followed at the same time‐points as those chosen for *Phytogbs* expression analysis. As reported in Fig. 3, three production peaks were detected: the first at 10 hpi during the first hours of the interaction between the plant and the bacteria, the second at 4 dpi during the early development of the nodule, and the third at 3–4 wpi when nodule reaches maturity. In view of the repetitions, a fourth peak is possible at 6 wpi, but this needs further investigation. When expressed as a function of protein mass (Fig. S6), the amount of NO production in the nodules is close to that in the roots, but the NO peak pattern remains the same. Such a pattern, which does not exclude the possibility of other production peaks on shorter time steps, highlights the fact that NO production presents wide fluctuations during the symbiotic process which coincide with the expression pattern of *Phytogb* genes, particularly *Phytog1.1*, suggesting their involvement in NO regulation.

### NO regulates phytoglobin gene expression

**Fig. 4:**
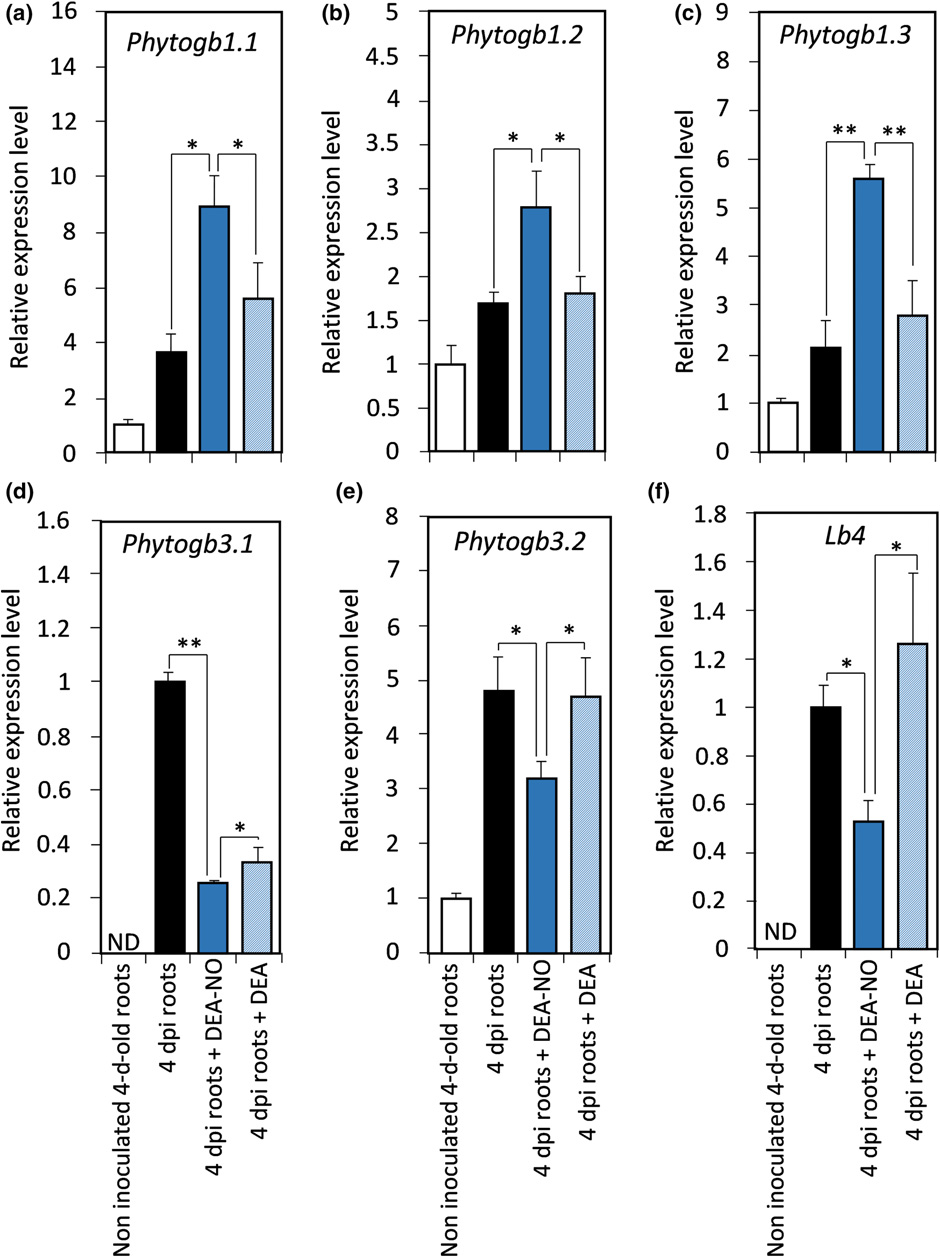
*Medicago truncatula Phytogb* genes expression after 4 d of nitric oxide (NO) donor treatment. Plant roots were either inoculated with *Sinorhizobium meliloti* in the presence or absence of 0.5 mM diethylamine (DEA)/diethylamine NONOate (DEA‐NO), or not inoculated (control), and grown for 4 d before RNA extraction and analysis. Data are means ± SE (*n* = 3). Each measurement was done in triplicate. *, *P* < 0.05; **, *P* < 0.01, according to the Student's *t*‐test. dpi, day post‐inoculation; ND, not detected.

Previous reports showed that *Phytogb1* genes are responsive to NO in *L. japonicus* and *Alnus firma* (Shimoda *et al.*, 2005; Sasakura *et al.*, 2006; Bustos‐Sanmamed *et al.*, 2011), but information was missing for most of the other *Phytogb* genes. To fill this gap, the effects of NO were analyzed on *M. truncatula Phytogb* gene expression in roots inoculated with *S. meliloti* and treated for 4 d with 0.5 mM of either the NO‐donor DEA‐NO, or its control DEA. DEA‐NO treatment was found to upregulate the expression of *Phytogb1.1*, *Phytogb1.2* and *Phytogb1.3* genes (Fig. 4a–c). As a positive control, the effects of DEA‐NO and DEA were analyzed on two plant defense marker genes, glutathione S‐transferase (*MtGST*, Medtr7g071380) (Gullner *et al*., 2018) and chalcone synthase (*MtCS*, Medtr1g124600) (Dao *et al.*, 2011), which are induced by NO in 4 dpi roots (Boscari *et al.*, 2013). Their induction in response to DEA‐NO confirmed the efficiency of the treatment (Fig. S7). Conversely, DEA‐NO treatment was found to downregulate the expression of *Phytogb3.1*, *Phytogb3.2* and *Lb4* genes (Fig. 4d–f). These results indicate that the six *Phytogb* genes are responsive to high NO concentration. Considered together, the data dealing with NO production pattern (Fig. 3), *Phytogb* expressions (Fig. 1) and NO‐donor effects (Fig. 4) indicate that a close relationship exists between NO concentration and *Phytogb1.1* expression.

### *Phytogb1.1* expression regulates NO concentration during symbiosis establishment and nodule organogenesis

**Fig. 5:**
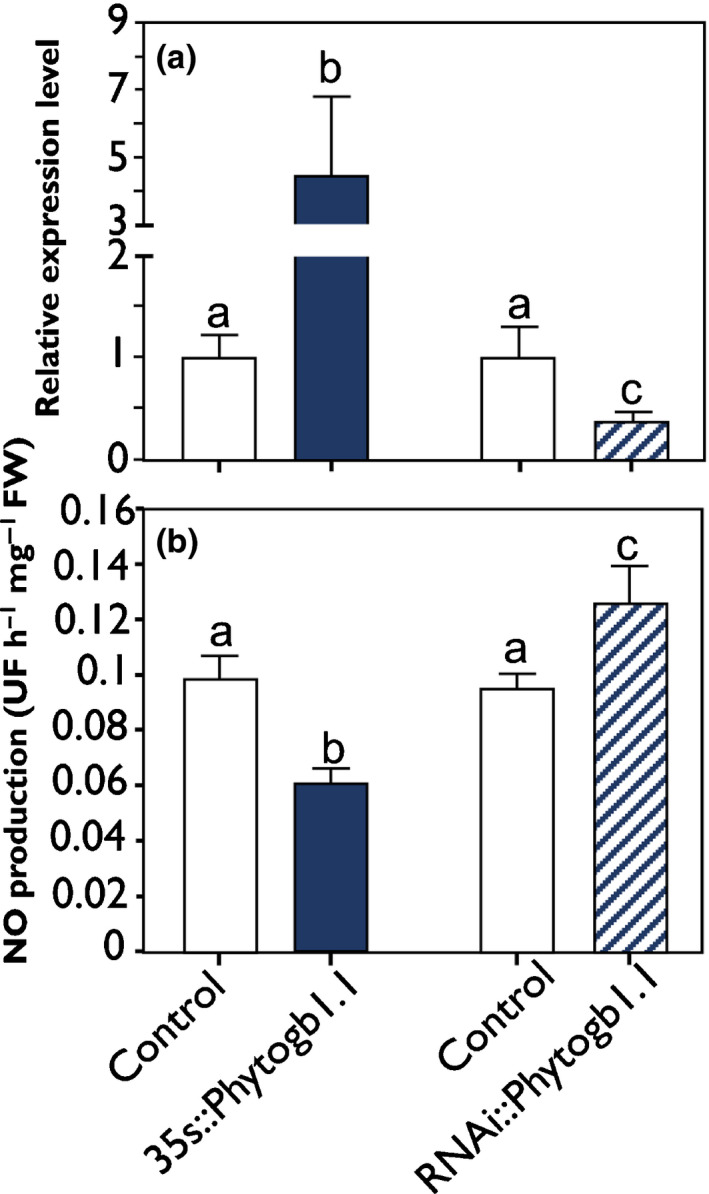
Relative expression level of *Medicago truncatula Phytogb1.1* and nitric oxide (NO) concentration in control and *Phytogb1.1*‐transformed roots at 4 d post‐inoculation (dpi). (a, b) Expression analysis of *Phytogb1.1* genes (a) and analysis of NO concentration (b) in control and transformed plant roots either overexpressing (35s::*Phytogb1.1*) or silencing *Phytogb1.1* (RNAi::*Phytogb1.1*) at 4 dpi. Data are means ± SE (*n* = 3). Each measurement was done in triplicate. Values followed by different letters are significantly different according to one‐way ANOVA analysis followed by a Fisher test (*P* < 0.05). UF, unit of fluorescence.

To analyze the potential involvement of Phytogb1.1 in the modulation of NO concentration during early symbiosis steps, we generated two types of *M. truncatula* transformed roots. 35s::*Phytogb1.1* overexpressed *Phytogb1.1*, and RNAi::*Phytogb1.1* silenced *Phytogb1.1* expression, both under the control of the constitutive CaMV 35s promoter (Fig. S8). At 4 dpi, 35s::*Phytogb1.1* roots showed a 4.5‐fold enhanced expression of *Phytogb1.1* as compared with control plants, whereas RNAi::*Phytogb1.1* roots showed a 2.5‐fold decrease in *Phytogb1.1* expression (Fig. 5a). The expression of *Phytogb 1.2, 1.3, 3.1* and *3.2* genes was not modified in *35s::Phytogb1.1* and RNAi*::Phytogb1.1* roots (Fig. S9a). NO concentrations were decreased 1.6‐fold and increased 1.3‐fold in 35s::*Phytogb1.1* and RNAi::*Phytogb1.1* roots, respectively (Fig. 5b), confirming that Phytogb1.1 regulates the concentration of NO in inoculated roots. No growth phenotype was visible on the transformed roots.

Then, the role of Phytogb1.1 in the nodulation process was investigated. As reported in Table 2, the nodule number per plant was lower both in 35s::*Phytogb1.1* and in RNAi::*Phytogb1.1* roots compared with control roots (Table 2). Interestingly, the treatment of nontransformed roots with 0.5 mM DEA‐NO during the 4 d following inoculation also resulted in a decreased nodule number per plant at 14 dpi without any other visible phenotype change on plant growth (Table 3). This indicates that both an excess and a lack of NO (± 30%) result in the inhibition of nodule establishment.

**Table 2:** Number of nodules in control and *Phytogb1.1*‐transformed *Medicago truncatula* plants at 14 d post‐inoculation (dpi).

Construct	Nodule number per plant
Control 35s	14.3 ± 1.1 a
*35s::Phytogb1.1 *	6.7 ± 0.5 b
Control RNAi	13.9 ± 0.8 a
*RNAi::Phytogb1.1 *	6.6 ± 0.4 b

Data are means ± SE (*n* = 3). Each measure was done with 12–18 plants. Values followed by different letters are significantly different according to one‐way ANOVA analysis followed by a Fisher test (*P* < 0.05).

**Table 3:** Number of *Medicago truncatula* nodules after 4 d of nitric oxide (NO) donor treatment.

Condition	Nodule number per plant
Control	7.7 ± 0.4 a
DEA‐NO	5.6 ± 0.5 b
DEA	7.8 ± 0.3 a

Plant roots inoculated with *Sinorhizobium meliloti* were treated with either 0.5 mM diethylamine NONOate (DEA‐NO) or 0.5 mM diethylamine (DEA). After 4 d, plants were transferred to a NO donor free medium and grown for an additional 10 d before measurement of nodule number. Data are means ± SE (*n* = 3). Each measure was done with more than 50 plants. Values followed by different letters are significantly different according to one‐way ANOVA analysis followed by a Fisher test (*P* < 0.05).

**Fig. 6:**
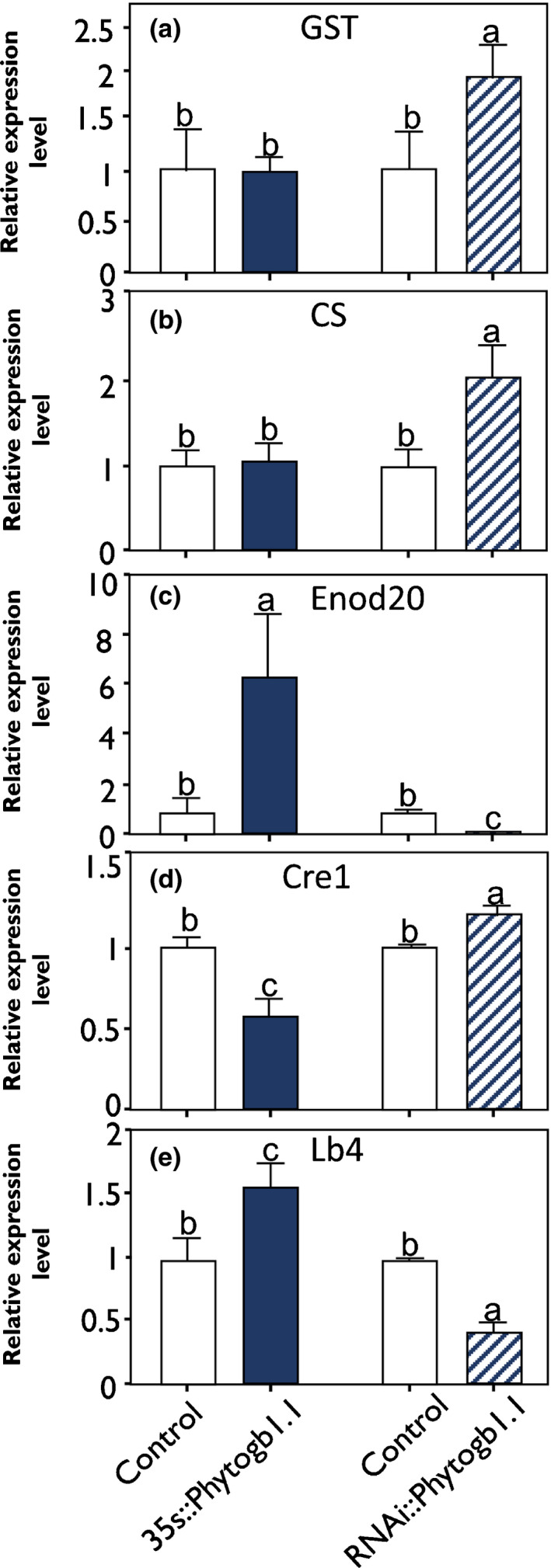
Relative gene expression level in control and *Phytogb1.1 Medicago truncatula*‐transformed roots at 4 d post‐inoculation (dpi). (a–e) Expression analysis of *GST* (a), *CS* (b), *Enod20* (c), *Cre1* (d), and *Lb*4 genes (e) in control and transformed plant roots either overexpressing (35s::*Phytogb1.1*) or silencing *Phytogb1.1*(RNAi::*Phytogb1.1*) at 4 dpi. Data are means ± SE (*n* = 3). Each measurement was done in triplicate. Values followed by different letters are significantly different according to one‐way ANOVA analysis followed by a Fisher test (*P* < 0.05).

To explore further the role of Phytogb1.1 and NO in the early stages of symbiosis, we analyzed the expression of various marker genes in control and transformed roots at 4 dpi. Both *GST* and *CS* genes were found to be induced in RNAi::*Phytogb1.1*, while their expression was unchanged in 35s::*Phytogb1.1* (Fig. 6a,b), indicating that their expression, and consequently the plant defense response, is upregulated by increased NO concentration, but not repressed under low NO. *Enod20* is a marker of rhizobia infection (Greene *et al*., 1998; Vernoud *et al.*, 1999). Its expression was upregulated in 35s::*Phytogb1.1* roots, and downregulated in RNAi::*Phytogb1.1* roots (Fig. 6c), indicating that the infection process is negatively regulated by NO. *Cre1* is a marker of nodule organogenesis (Frugier *et al.*, 2008). Its expression was downregulated in 35s::*Phytogb1.1* roots and upregulated in RNAi::*Phytogb1.1* roots (Fig. 6d), indicating a positive regulation of organogenesis by NO. *Lb4* was chosen as a representative marker of early nodule development and N_2_‐fixation machinery acquisition (Appleby, 1992). Its expression was higher in 35s::*Phytogb1.1* and lower in RNAi::*Phytogb1.1* compared with the control roots, supporting the idea that the early development of the nodule may be negatively regulated by NO (Fig. 6e).

### *Phytogb1.1* expression regulates NO concentration and nitrogen fixation in mature nodules

**Fig. 7:**
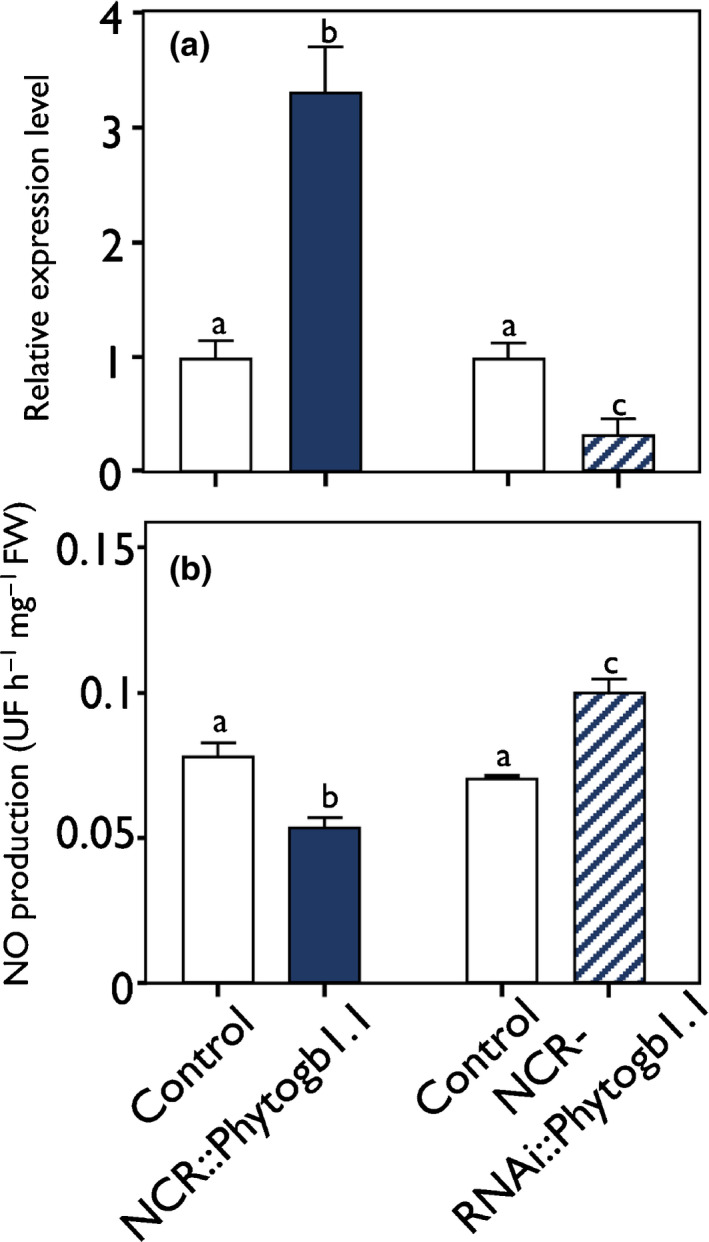
Relative expression level of *Medicago truncatula Phytogb1.1* and nitric oxide (NO) concentration in 3 wk post‐inoculation (wpi) nodules of control and *Phytogb1.1*‐transformed plants. (a, b) Expression analysis of *Phytogb1.1* gene (a) and analysis of NO concentration (b) in control and transformed plant nodules either overexpressing (NCR::*Phytogb1.1*) or silencing *Phytogb1.1 *(NCR‐RNAi::*Phytogb1.1*) at 3 wpi. Data are means ± SE (*n* = 3). Each measurement was done in triplicate. Values followed by different letters are significantly different according to one‐way ANOVA analysis followed by a Fisher test (*P* < 0.05). UF, unit of fluorescence.

To analyze the role of *Phytogb1.1* specifically in mature nodules, new constructions either overexpressing (NCR::*Phytogb1.1*) or silencing (NCR‐RNAi::*Phytogb1.1*) *Phytogb1.1* were designed using the nodule zone III specific promoter NCR001 (Mergaert *et al.*, 2003). These constructs present the advantage of modifying the expression of *Phytogb1.1* in the N_2_‐fixing zone without impacting the formation and development of the nodule. The result was that *Phytogb1.1* was 3.3‐fold more and three‐fold less expressed in 3 wpi NCR::*Phytogb1.1* and NCR‐RNAi::*Phytogb1.1* nodules, respectively, compared with their respective controls (Fig. 7a). The expression of *Phytogb 1.2*, *1.3*, *3.1* and *3.2* genes was not modified in NCR*::Phytogb1.1* and NCR‐RNAi*::Phytogb1.1* nodules (Fig. S9b). NO concentrations were 1.5‐fold decreased and 1.4‐fold increased in the NCR::*Phytogb1.1* and the NCR‐RNAi::*Phytogb1.1* nodules, respectively, compared with their control (Fig. 7b), indicating that Phytogb1.1 regulates the concentration of NO in mature nodules. No growth phenotype was visible on the transformed roots.

The functional state of 3 wpi nodules was assessed through the measurement of the *in vivo* nitrogenase activity (measured as ARA) to evaluate their N_2_‐fixing capacity and the ATP : ADP ratio to evaluate their energy state (Table 4). Compared with their control, NCR*::Phytogb1.1* nodules exhibited a 34% higher ARA, while RNAi‐NCR::*Phytogb1.1* ones exhibited a 30% reduced ARA. Similarly, when compared with control nodules, ATP : ADP ratios were found to be higher in NCR*::Phytogb1.1* nodules (8.0 ± 0.3) and lower in RNAi‐NCR::*Phytogb1.1* nodules (4.8 ± 0.1). These data clearly indicate that Phytogb1.1 is able to modulate the energy and N metabolism of mature nodules, presumably through the regulation of the concentration of NO.

**Table 4:** Nitrogenase activity and energy state in control and *Phytogb1.1*‐transformed *Medicago truncatula* nodules at 3 wk post‐inoculation (wpi).

Construct	ARA (nmol ethylene h^−1^ mg^−1^ nodule)	ATP : ADP ratio
Control 35s	15.8 ± 1.1 a	6.9 ± 0.1 a
*35s::Phytogb1.1 *	21.2 ± 1.4 b	8.1 ± 0.3 b
Control RNAi	16.7 ± 1.6 a	7.0 ± 0.1 a
*RNAi::Phytogb1.1 *	11.0 ± 0.8 c	4.8 ± 0.1 c

Nitrogenase activity (estimated as ARA) was normalized per nodule FW. Energy state was measured as ATP : ADP ratio. Data are means ± SE (*n* = 3). Each measurement was done in triplicate. Values followed by different letters are significantly different according to one‐way ANOVA analysis followed by a Fisher test (*P* < 0.05).

**Fig. 8:**
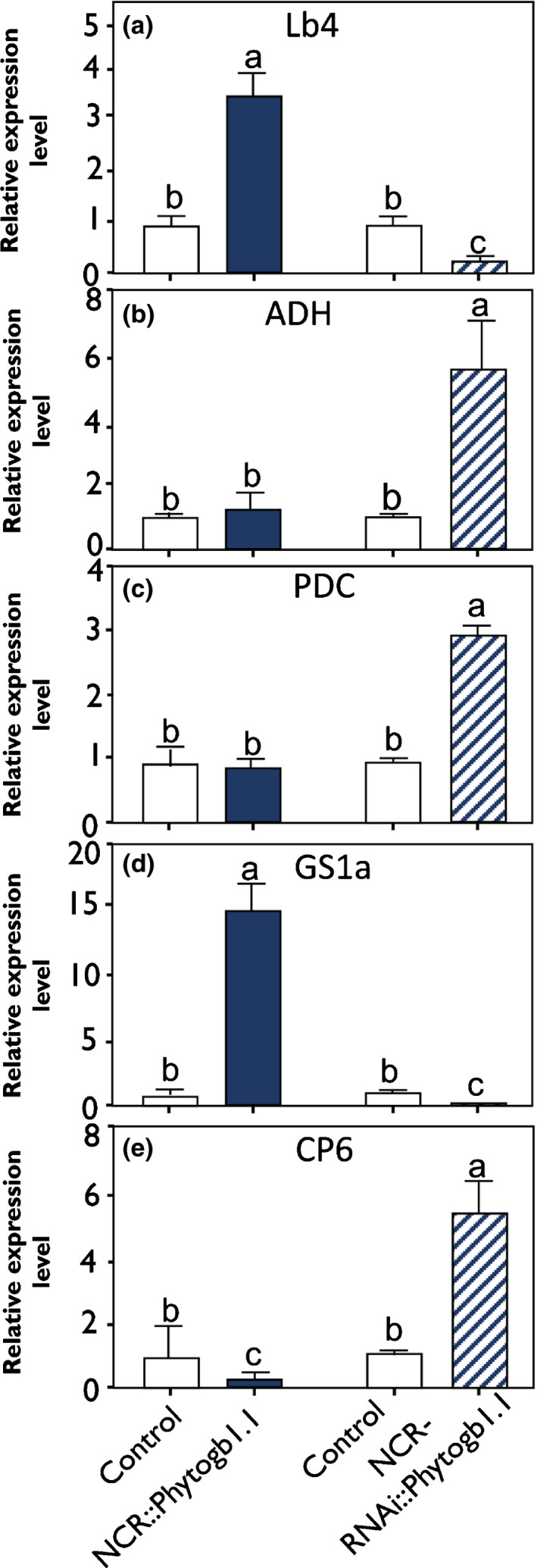
Relative gene expression level in 3 wk post‐inoculation (wpi)‐old nodules of control and Phytogb1.1 *Medicago truncatula* transformed plants. (a–e) Expression analysis of *Lb4* (a), *ADH *(b), *PDC *(c), *GS1a *(d), and *CP6* genes (e) in control and transformed plant nodules either overexpressing (NCR::*Phytogb1.1*) or silencing *Phytogb1.1* (NCR‐RNAi*::Phytogb1.1*) at 3 wpi. Data are means ± SE (*n* = 3). Each measurement was done in triplicate. Values followed by different letters are significantly different according to one‐way ANOVA analysis followed by a Fisher test (*P* < 0.05).

To go further in the understanding of the role of Phytogb1.1 in these processes, the expression of genes involved in N_2_‐reduction and assimilation, hypoxia and senescence was analyzed in control and transformed nodules (Fig. 8). The expression of glutamine synthetase 1a (GS1a), involved in the assimilation of N (Groat & Vance, 1981), and of *Lb4* was found to be clearly induced in NCR::*Phytogb1.1* nodules and reduced in NCR‐RNAi::*Phytogb1.1* nodules as compared with their respective controls (Fig. 8a,b), indicating a negative regulation of N_2_ fixation by NO. As mature nodules exhibit a microoxic environment, the expression of alcohol dehydrogenase (*ADH*) and pyruvate decarboxylase (*PDC*), two marker genes of hypoxia (Bailey‐Serres & Voesenek, 2008), was analyzed. Their expression was increased in NCR‐RNAi::*Phytogb1* nodules, whereas it was unchanged in NCR::*Phytogb1.1* nodules(Fig. 8c,d), indicating that a rise of NO concentration in the nodule activates the expression of hypoxia‐responsive pathway. Finally, we analyzed the expression of the cysteine protease 6 (*CP6*) gene, a reliable marker of senescence in *M. truncatula* nodules (Van de Velde *et al.*, 2006; Pierre *et al.*, 2014). *CP6* was found to be downregulated in NCR::*Phytogb1.1* nodules, and strongly upregulated in NCR‐RNAi::*Phytogb1.1* nodules (Fig. 8e), indicating that overexpression of *Phytogb1.1* delayed the senescence, while its downregulation promoted it.

## Discussion

In this study we identified 17 *M. truncatula Phytogb* genes composed of 12 *Lb*, three *Phytogb1* and two *Phytogb3*. *Lbs* are close to each other, but phylogenetically (Fig. S1) and structurally (Fig. S2) different from the other *Phytogb* types (Vinogradov *et al.*, 2006). Most *MtLbs* are strongly expressed in the nodule interzone II–III, whereas *MtLb2* is equally expressed in the interzone and zone III, and *MtLb3* is mainly expressed in zone III (Table S1), confirming that several Lb classes exist with different locations within the nodule. The role of Lb diversity in legumes plants is not fully figured out, even if it was proposed that, in addition to their role as oxygen‐carriers (Appleby, 1992), the abundance of Lbs could be one of the cornerstones necessary for the functioning of a Phytogb–NO cycle in microaerobic conditions, such as that prevailing in nodules (Hichri *et al.*, 2015; Berger *et al.*, 2019).

The main objective of this study was to analyze the expression pattern of the different *Phytogbs* and the NO production throughout the symbiotic process, and to identify the Phytogbs potentially involved in NO regulation. Our results show that three Phytogb expression and NO production peaks can be considered (Figs 1, 2, 3, 4): during the first hours of the symbiotic interaction (10 hpi); during the early development of the nodule (4 dpi); and when the nodule becomes mature (3–4 wpi). The most salient feature emerging from this analysis is the high expression of *Phytogb1.1*, which fits particularly with NO variation pattern from the beginning of the interaction to the N_2_‐fixing nodule step (Figs 1, 2, 3). This led us to generate *Phytogb1.1*‐overexpressing and ‐silencing plants to investigate in greater detail the connection between Phytogb1.1 and NO during the symbiotic process.

### The Phytogb1.1‐NO couple regulates symbiosis establishment and nodule organogenesis

The ‘Phytogb1‐NO’ couple forms a feedback loop that allows NO concentration to be quickly regulated (Hill, 2012). Here, we demonstrate that the overexpression of *Phytogb1.1* decreases NO production while its silencing increases it (Fig. 5b), confirming that Phytogb1.1 negatively regulates NO concentration as previously reported in *L. japonicus* (Nagata *et al.*, 2008; Shimoda *et al.*, 2009; Fukudome *et al.*, 2016). Both higher and lower NO concentrations inhibit the nodulation (Table 2). These results are consistent with a previous report showing that high NO concentration inhibits the nodulation by affecting the formation of an infection thread (Fukudome *et al.*, 2016), but they also confirm the observations that nodulation is inhibited by a decrease in NO concentration (Pii *et al*., 2007; del Giudice *et al.*, 2011). This means that an excess as well as a lack of NO impair nodule establishment and growth, and that NO concentration needs to be tightly regulated at the site of nodule initiation for a successful establishment of the symbiotic relationship.

Based on the feedback mechanism of the Phytogb1.1–NO couple, the first transitory NO production peak observed at 10 hpi (Fig. 3) may be linked to the sharp and transient decrease in *Phytogb1.1* at 4 hpi (Fig. 1). The subsequent upregulation of *Phytogb1.1* at 10 hpi may be linked to the NO production peak as observed in the *L. japonicus* root surface when inoculated with its symbiont *Mesorhizobium loti* (Nagata *et al.*, 2008). This NO peak may be related to the defense mechanisms established by the plant in response to the rhizobium. In *G. max* (Libault *et al*., 2010), *L. japonicus* (Stacey *et al*., 2006) and *M. truncatula* (Jones *et al*., 2008) roots, a large number of plant defense genes have been shown to be induced within 12 hpi with their symbiotic rhizobia, and their expression gradually returned to background levels within 24 hpi when the infection process was initiated. Increased *GST* and *CS* gene expression in both RNAi::*Phytogb1.1 M. truncatula* roots (Fig. 6) and roots treated with NO‐donor (Fig. S7) were in agreement with the literature and indicate that the induction of plant defense mechanisms is linked to higher NO concentration resulting from *Phytogb1.1* downregulation.

The upregulation of *Phytogb1.1* at 10 hpi triggers the decrease in NO concentration to its basal value for 2 d (Fig. 3). In *M. truncatula*, *Enod20*, a marker of root infection and cortical cell activation, was shown to be mainly expressed during the formation of the infection thread and the initiation of the nodule primordium (Greene *et al*., 1998; Vernoud *et al*., 1999) which take place between 1 and 3 dpi (Timmers *et al*., 1999; Xiao *et al.*, 2014). In 35s::*Phytogb1.1* roots with low NO concentration, *Enod20* is highly expressed (Fig. 6c), whereas it is weakly expressed in RNAi::*Phytogb1.1* roots with high NO concentration. These observations indicate that the infection of the plant and the activation of cortical cells require a low NO concentration and a decreased plant defense response. This explanation is consistent with observations made in a NR‐deficient double mutant *A. thaliana* plant line (Vitor *et al*., 2013). This mutant, exhibiting a low NO concentration, is prone to infection by pathogens.

The second NO production peak observed at 4 dpi (Fig. 1) suggests that NO is involved in the onset of nodule organogenesis that starts from 3 to 4 dpi in the *M. truncatula–S. meliloti* symbiosis (Oldroyd & Downie, 2008; Xiao *et al.*, 2014). Such an involvement is consistent with the observation that, in 4 dpi *M. truncatula* roots, NO scavenging resulted in the downregulation of many cell division and growth‐related genes (Boscari *et al.*, 2013). *Cre1*, which encodes for a cytokinin receptor, regulates the symbiotic interaction and is considered as a nodule organogenesis marker upregulated by NO (Ferrarini *et al.*, 2008; Frugier *et al*., 2008; del Giudice *et al.*, 2011). During the first days following inoculation with symbiotic rhizobia, a specific production of NO was reported in the pericycle, endodermis and dividing cortical root cells, where *Cre1* is expressed and the nodule primordium is initiated (del Giudice *et al.*, 2011; Plet *et al*., 2011). The induction and repression of *Cre1* in RNAi::*Phytogb1.1* and 35S::*Phytogb1.1* roots (Fig. 6c), respectively, means that high NO promotes nodule development, while low NO inhibits it. This suggests that *Cre1* induction and the onset of nodule organogenesis are under the control of NO and Phytogb1.1. Lbs, whose expression in young developing *M. truncatula* nodules starts strongly from 5 dpi (Gallusci *et al*., 1991), are markers of N_2_ fixation (Appleby, 1992). Our results show that the high expression of *Lb4*‐*Lb3* after 4 dpi (Fig. 1) is correlated with a decrease in NO concentration between 4 and 14 dpi (Fig. 3), and that NO represses the expression of *Lb4* (Figs 4, 6). This means that after the onset of nodule organogenesis, a decrease in NO concentration is necessary for the development and growth of the nodule.

Considered together, our results led us to propose a scenario in which the Phytogb1.1–NO couple plays a role of symbiosis regulator. First, within hours after inoculation, the low level of *Phytogb1.1* (4 hpi) allows an increase of NO. The increase in NO concentration (at 4 and 10 hpi) allows the establishment of plant defense reactions (induction of *GST*, *CS*) as well as the induction of *Phytogb1.1* (10 hpi). Second, increased Phytogb1.1 activity reduces NO to its initial concentration (1–2 dpi), which, in turn, lowers the defense reactions, allowing the infection and the reception of the symbiont (induction of *Enod20*) and subsequently downregulation of *Phytogb1.1* expression (1–2 dpi). Third, low Phytogb1.1 triggers a new rise in NO concentration (between 2 and 4 dpi) which allows the initiation of nodule organogenesis (induction of *Cre1*) and, again, the induction of *Phytogb1.1* (4 dpi). Finally, once the organogenesis is initiated, the subsequent decrease in NO concentration (between 9 and 14 dpi) accompanies the nodule development and growth (induction of *Lb4*). It is therefore easy to understand the reduction in nodule number under both high and low NO concentrations (Tables 2, 3): a high NO concentration inhibits the infection process, whereas a low NO concentration inhibits nodule organogenesis.

A recent study on mycorrhizal symbiosis between *Solanum lycopersicum* and *Rhizophagus irregularis* also shows two peaks of NO production, in the hours following inoculation and then at 48 hpi, under the control of SlPhytogb1 (Martinez‐Medina *et al.*, 2019). Such similar behavior suggests that the establishment of the interaction and the symbiotic organogenesis are controlled by the Phytogb1.1–NO couple in both N_2_‐fixing and mycorrhizal symbiosis.

### The Phytogb1.1–NO couple modulates energy and N_2_‐fixing metabolism

At 3–4 wpi, nodules reach their mature N_2_‐fixing state. This period is characterized by a strong and a moderate increase in *Lbs* and *Phytogb1.1* expression (Figs 1, 2), respectively, and a high NO concentration (Fig. 3). The question of Lbs and Phytogb1.1 functions with regard to NO may be raised. The presence of Lb–NO complexes, detected by electron paramagnetic resonance (EPR), in soybean and *L. japonicus* nodules *in vivo* (Maskall *et al.*, 1977; Mathieu *et al.*, 1998; Sánchez *et al*., 2010) shows that Lbs are involved in the complexation of NO. It may be noted that the higher level of *Lb* gene expression observed in the interzone II–III rather than in zone III (Table S1) is consistent with the fact that NO represses *Lb* gene expression (Fig. 4) and that it is mainly produced in zone III (Baudouin *et al.*, 2006). The ability of Lbs to bind O_2_ and NO to produce NO_3_^–^ (Herold & Puppo, 2005) makes them good candidates to detoxify NO which is present in high concentrations in the mature nodules (Baudouin *et al.*, 2006).

Although less expressed than *Lbs*, the significant expression of *Phytogb1.1* in nodules (Fig. 2) suggests that Phytogb1.1 has its own function in the N_2_‐fixing metabolism. In *L. japonicus* nodules*, LjHb1*overexpression results in decreased NO content and increased ARA (Shimoda *et al.*, 2009; Fukudome *et al*., 2019), whereas *LjGlb1.1* mutants nodules show higher NO content and lower ARA (Fukudome *et al.*, 2016). These authors suggested that the role of *Phytogb1.1* is to scavenge NO to avoid the inhibition of the nitrogenase and the N_2_ fixation. Here, lower NO concentration in *Phytogb1.1*‐overexpressing nodules resulted in higher *Lb4* and *GS1a* expression, and higher ARA and energy state, while opposite effects were observed in NCR‐RNAi*::Phytogb1.1* nodules (Figs 7, 8; Table 4), indicating that high NO concentration inhibits N_2_‐fixing metabolism, whereas low NO concentration favors it. This regulation occurs both at post‐translational and transcriptional levels. First, NO is a potent inhibitor of nitrogenase (Trinchant & Rigaud, 1982; Kato *et al.*, 2010) and disrupts Lb and GS1a activities after nitration of their tyrosine moieties by peroxynitrite, a NO derivative (Melo *et al*., 2011; Navascues *et al.*, 2012). At the gene level, NO represses the expression of the bacterial *nifH* and *nifD* in soybean nodules (Sánchez *et al*., 2010), and present data show that it also represses the expression of key genes in the N_2_‐fixing metabolism (*Lb4* and *GS1a*).

However, it should be noted that ARA is more substantial in *L. japonicus* nodules in the presence of 0.1 mM single nucleotide polymorphism (SNP; NO donor) than in either the absence or presence of higher (1 mM) concentrations of SNP, indicating that low but significant NO concentration is beneficial to N_2_ fixation (Kato *et al.*, 2010). The microoxic environment prevailing in nodules raises the question of energy supply. Accumulated data support the functioning of Phytogb–NO respiration in nodules (Horchani *et al.*, 2011). Both Lbs and Phytogb1.1 have the ability to bind O_2_ and NO to produce NO_3_^−^ (Herold & Puppo, 2005), which makes them good candidates to participate in the regeneration of ATP through the functioning of the Phytogb–NO respiration. In the present study, the silencing of *Phytogb1.1* and the increase in NO concentration trigger the overexpression of *ADH* and *PDC* (Fig. 8), which mimics a situation of hypoxia. The decrease in ATP : ADP ratio and ARA in *Phytogb1.1*‐silenced nodules, and their increase in *Phytogb1.1*‐overexpressing nodules (Table 4) indicate that Phytogb1.1 participates in NO turnover, but is also involved (alongside Lb?) in the functioning of Phytogb–NO respiration and the maintenance of the nodules’ energy state. The very recent elucidation of the role of NO and Phytogb1 in the perception of hypoxia in *A. thaliana* (Hartman *et al.*, 2019) makes it possible to hypothesize that the Phytogb1.1–NO couple is also involved in the regulation of the nodule metabolism. This hypothesis is a promising challenge for future investigations.

**Fig. 9:**
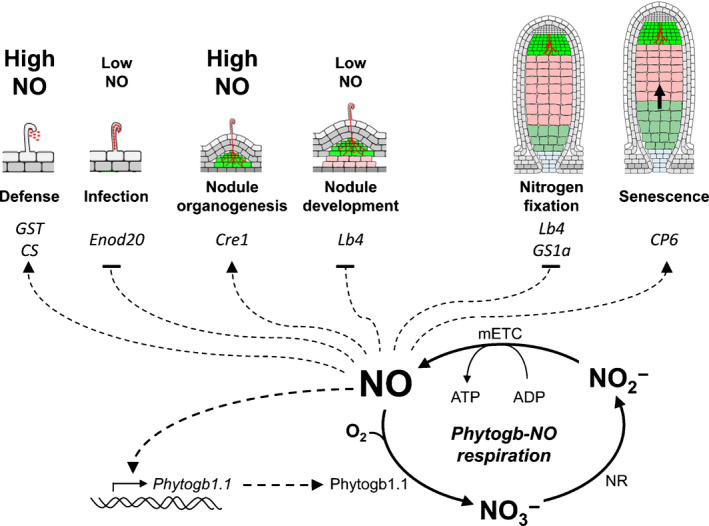
Synoptic representation of Phytogb1.1 and nitric oxide (NO) function during the symbiotic process. Depending on the steps of the symbiotic process, the NO concentration either increases (high NO) or decreases (low NO) and regulates the transition between the different stages of the symbiosis. The NO content is regulated by the Phytogb1.1‐NO loop. In microoxic mature nodules, Phytogb–NO respiration regulates the concentration of NO and ensures the regeneration of energy (ATP) necessary for the functioning of plant cell metabolism. The arrow indicates the direction of increase of senescent tissue. mETC, mitochondrial electron transfer chain; NR, nitrate reductase.

In conclusion, this work highlights the regulatory role of Phytogb1.1 in the regulation of NO during the early stages of symbiosis (defense response, infection, nodule organogenesis), and in the N_2_‐fixing nodule, as well as the close relationship between NO production and the expression of the other Phytogb genes (Fig. 9). However, the control of NO in the nodule cannot be done only by the plant partner. Indeed, the *S. meliloti* flavohemoglobin was shown to be involved in NO degradation and is essential in maintaining efficient N‐fixing symbiosis (Meilhoc *et al.*, 2010; Cam *et al.*, 2012). Otherwise, the bacterial NO reductase and the nnrS system were also shown to regulate NO concentration in N_2_‐fixing nodules (Meilhoc *et al.*, 2013; Blanquet *et al.*, 2015). How the regulatory systems of the plant and the bacterial partners are coordinated to control NO is one of the main issues to decipher the toxic, signaling, and metabolic functions of NO at each stage of the symbiotic interaction.

## Author contributions

ABerger, ABoscari and RB planned and designed the research. ABerger and SG performed the experiments. ABerger, ABoscari and RB analyzed the data. ABerger, ABoscari, AP and RB interpreted the data and wrote the manuscript.

## Supporting information

Please note: Wiley Blackwell are not responsible for the content or functionality of any Supporting Information supplied by the authors. Any queries (other than missing material) should be directed to the *New Phytologist* Central Office.

**Fig. S1 **Phylogenetic tree of *Medicago truncatula*, *Lotus japonicus*, *Glycine max* and *Arabidopsis thaliana* phytoglobins.
**Fig. S2** Multiple sequence alignment of *Medicago truncatula *phytoglobins.
**Fig. S3** Microarray data for *Medicago truncatula *phytoglobins.
**Fig. S4** (a) Chromosomal localization and (b) exon‐intron structure of *Medicago truncatula Phytogb *genes.
**Fig. S5** MEME model of primary sequences of *Medicago truncatula *Phytogb proteins.
**Fig. S6** Variations of NO concentration during the symbiotic process expressed as a function of protein content.
**Fig. S7** Defense gene (*GST *and *CS*) expression after 4 d of NO donor treatment.
**Fig. S8** Pictures of control, *35s::Phytogb1.1 *and *RNAi::Phytogb1.1 *transgenic hairy root growth on Petri dishes at 2 d after inoculation with *Sinorhizobium meliloti*.
**Fig. S9** Relative expression level of class 1 and 3 *Phytogb *genes in *Phytogb1.1*‐transformed roots.
**Table S1** Primer sequences for quantitative RT‐PCR analysis.
