# Supplementary material for: Medicago truncatula Phytoglobin 1.1 controls symbiotic nodulation and nitrogen fixation via the regulation of nitric oxide concentration
Source: New Phytol. 2020 Mar 14;227(1):84–98. doi: 10.1111/nph.16462 (PMC7317445; doi:10.1111/nph.16462)
Supplement: Supplementary file 1 — Fig. S1 Phylogenetic tree of Medicago truncatula, Lotus japonicus, Glycine max and Arabidopsis thaliana phytoglobins. Fig. S2 Multiple sequence alignment of Medicago truncatula phytoglobins. Fig. S3 Microarray data for Medicago truncatula phytoglobins. Fig. S4 (a) Chromosomal localization and (b) exon‐intron structure of Medicago truncatula Phytogb genes. Fig. S5 MEME model of primary sequences of Medicago truncatula Phytogb proteins. Fig. S6 Variations of NO concentration during the symbiotic process expressed as a function of protein content. Fig. S7 Defense gene (GST and CS) expression after 4 d of NO donor treatment. Fig. S8 Pictures of control, 35s::Phytogb1.1 and RNAi::Phytogb1.1 transgenic hairy root growth on Petri dishes at 2 d after inoculation with Sinorhizobium meliloti. Fig. S9 Relative expression level of class 1 and 3 Phytogb genes in Phytogb1.1‐transformed roots. Table S1 Primer sequences for quantitative RT‐PCR analysis. [file NPH-227-84-s001.pdf]

New Phytologist Supporting Information

Article title: *Medicago truncatula* Phytoglobin 1.1 controls symbiotic nodulation and nitrogen fixation via the regulation of nitric oxide level

Authors: Antoine Berger, Sophie Guinand, Alexandre Boscari, Alain Puppo, Renaud Brouquisse

Article acceptance date: 19 January 2020

Table S1: Primer sequences for quantitative RT-PCR analysis

Genes	Code gene Mt4.0	Description	Sequences 5'-3'	References
Mtc27	Medtr2g436620	housekeeping gene	F : GTGGGAGGTTGAGGGAAAGT; R : TTGAAGGTCCTTGAGCTTGC	del Guidice et al., 2011
a38	Medtr4g109650	housekeeping gene	F : TCGTGGTGGTGGTTATCAAA ; R : TTCAGACCTTCCCATTGACA	del Guidice et al., 2011
Enod20	Medtr8g145270	early noduline	F : TCAACTCCAATTCCTCATCC; R : AATCTGAAGGTGACGGTG	del Guidice et al., 2011
Cre1	Medtr8g106150	cytokinin receptor histidine kinase	F : CTCTTGCCATCCTTGTTTCAA; R : GTGCATAGGCCACTCCACTAA	del Guidice et al., 2011
Phytogb1.2	Medtr4g068870	no symbiotic hemoglobin	F : GGACAATGCCAATTTGATAAGCAG ; R : CTGGTGGAGCAATCTCAAGG	This work
Phytogb1.3	Medtr0026s0210	no symbiotic hemoglobin	F : TTCTCATGACATGTGAATCAGC; R : GTGACCACATTTCAGGTAATGC	This work
Lb 3	Medtr1g090810	leghemoglobin	F : GGACAATGCCAATTTGATAAGCAG ; R : CTGGTGGAGCAATCTCAAGG	This work
Lb 4	Medtr1g011540	leghemoglobin	F : GAGCGAAGAATTGAGCACTGCT ; R : TGCCTTCTTAATTGCAGTTGCC	This work
Phytogb3.1	Medtr3g109420	truncated hemoglobin	F : GCTTCATCACACACACATAC ; R : AATCATGATCTATATCTGAAATGTT	This work
Phytogb3.2	Medtr1g008700	truncated hemoglobin	F : AACTTTATAAGTTTTCTTTTGTTTG ; I R : GATAGACATATAGACGTTCAATCTT	This work
GS1	Medtr3g065250	glutamine synthetase isoform 1	F : CTTGACCTCTCCGAAACCA; R : CTTGGGAAGCTGTGAAGGG	This work
CP6	Medtr4g079800	cysteine protease	F : CCTGCTGCTACTATTGCTGGATATG; R : CACTCGCATCAATGGCTACGG	Pierre et al., 2014
GST	Medtr7g065600	glutathione S-transferase	F : TTTGTTCACTAGTGAGAAATTTCC; R : GAAGACTTTCATAACGAGCTTTAA	Boscari et al., 2013
CS	Medtr1g124600	chalcone synthase	F : AAAGA5TAAATCCACCAGAG; R : AAACACCAAACTCAAGTCCT	Boscari et al., 2013
ADH	Medtr3g089940	alcohol dehydrogenase	F : GGGACTATGTTCTCAATCTGG; R : TAGGTACCAAATGTCACAGTCTC	This work
PDC	Medtr2g015560	pyruvate decarboxylase	F : GCCCCGCGTTAAGATCAAC; R : CCAAGTTATTCACCACTGCCT	This work

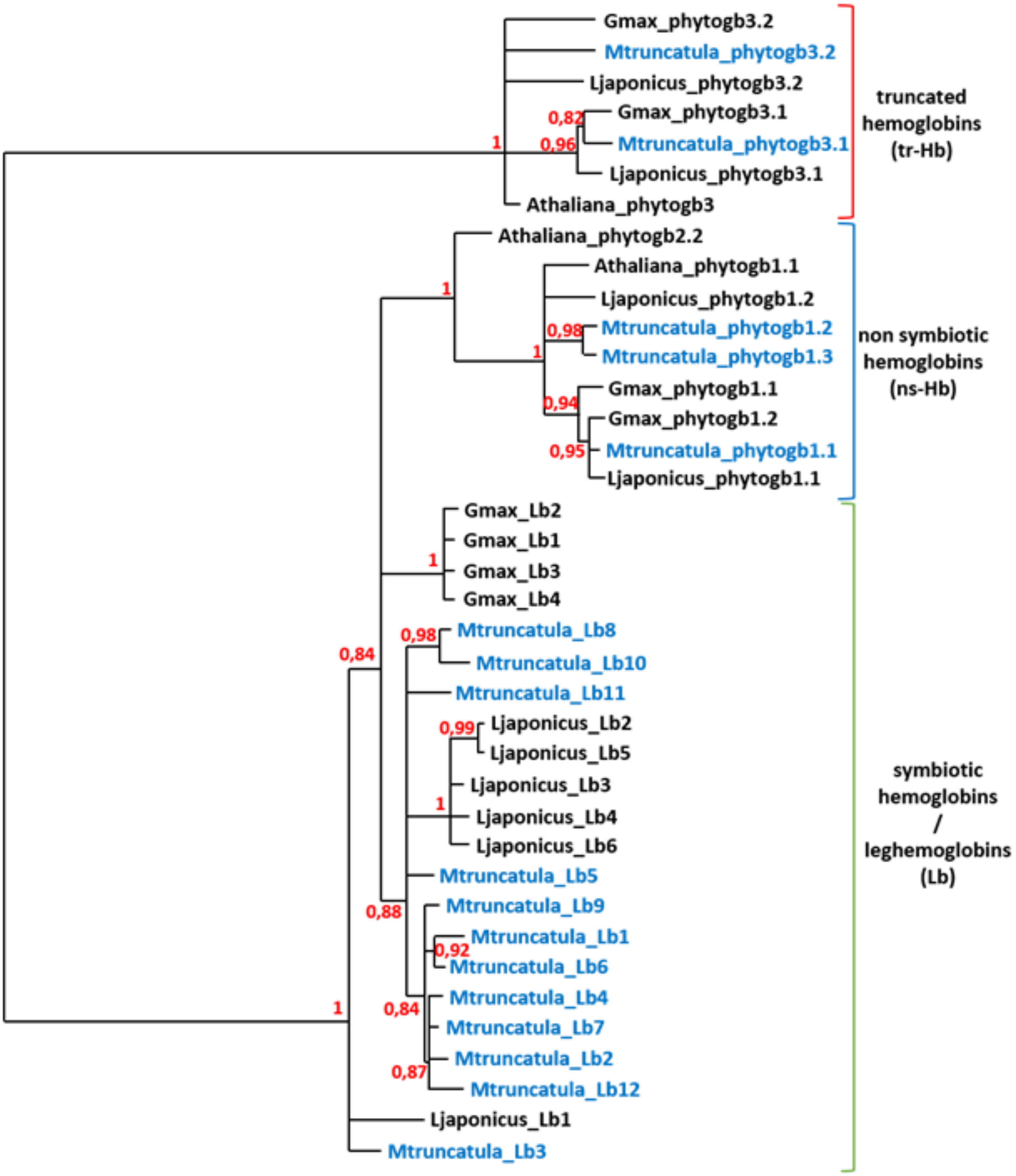

Figure S1: Phylogenetic tree of *Medicago truncatula, Lotus japonicus, Glycine max and Arabidopsis thaliana* phytoglobins.

Medicago Phytoglobin sequences were extracted from Noble database with Mt4.0 genome version. Phytoglobin sequences from *Glycin max*, *Lotus japonicus* and *Arabidopsis thaliana* were extracted from NCBI database. Phytoglobin protein sequences were aligned with MUSCLE program and the phylogenetic tree was constructed by maximum likelihood method using PhymL (http://www.atgc-montpellier.fr/phyml/). Nodes with bootstrap values less than 80% were collapsed into polytomies.

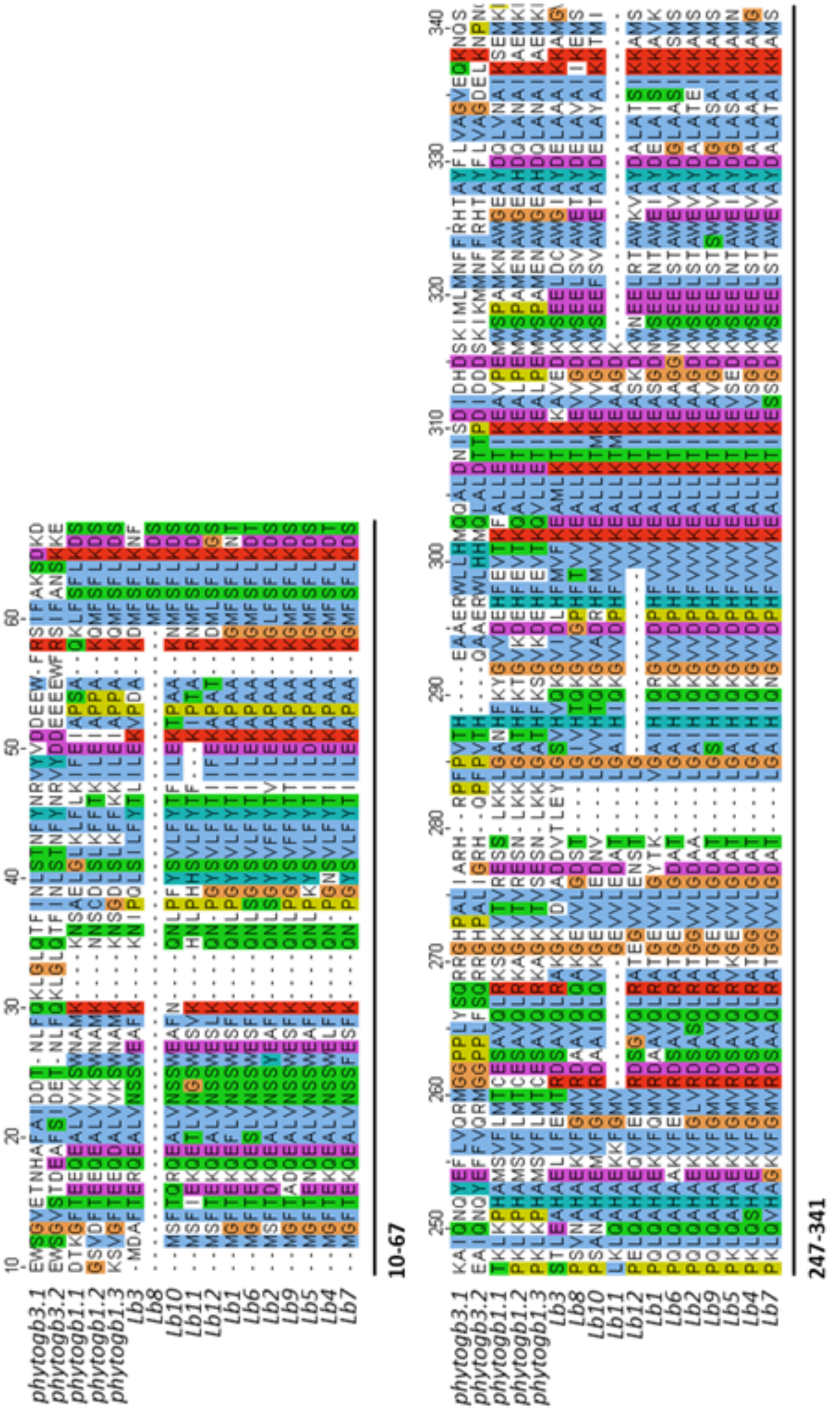

Figure S2: Multiple sequence alignment of *Medicago truncatula* Phytoglobins.

Alignments of *M. truncatula* phytoglobin sequences computed by Muscle (https://www.ebi.ac.uk/Tools/msa/muscle/). ClustalX color code was used to highlight the protein segment properties: hydrophobic residue (AILMFW) in blue, acidic residue (DE) in magenta, basic residue (RK) in red, polar residue (QSNT) in green, other aromatic residue (YH) in dark blue and specific color for CP and G. Figure was generated using Jalview 2.7 (http://www.jalview.org).

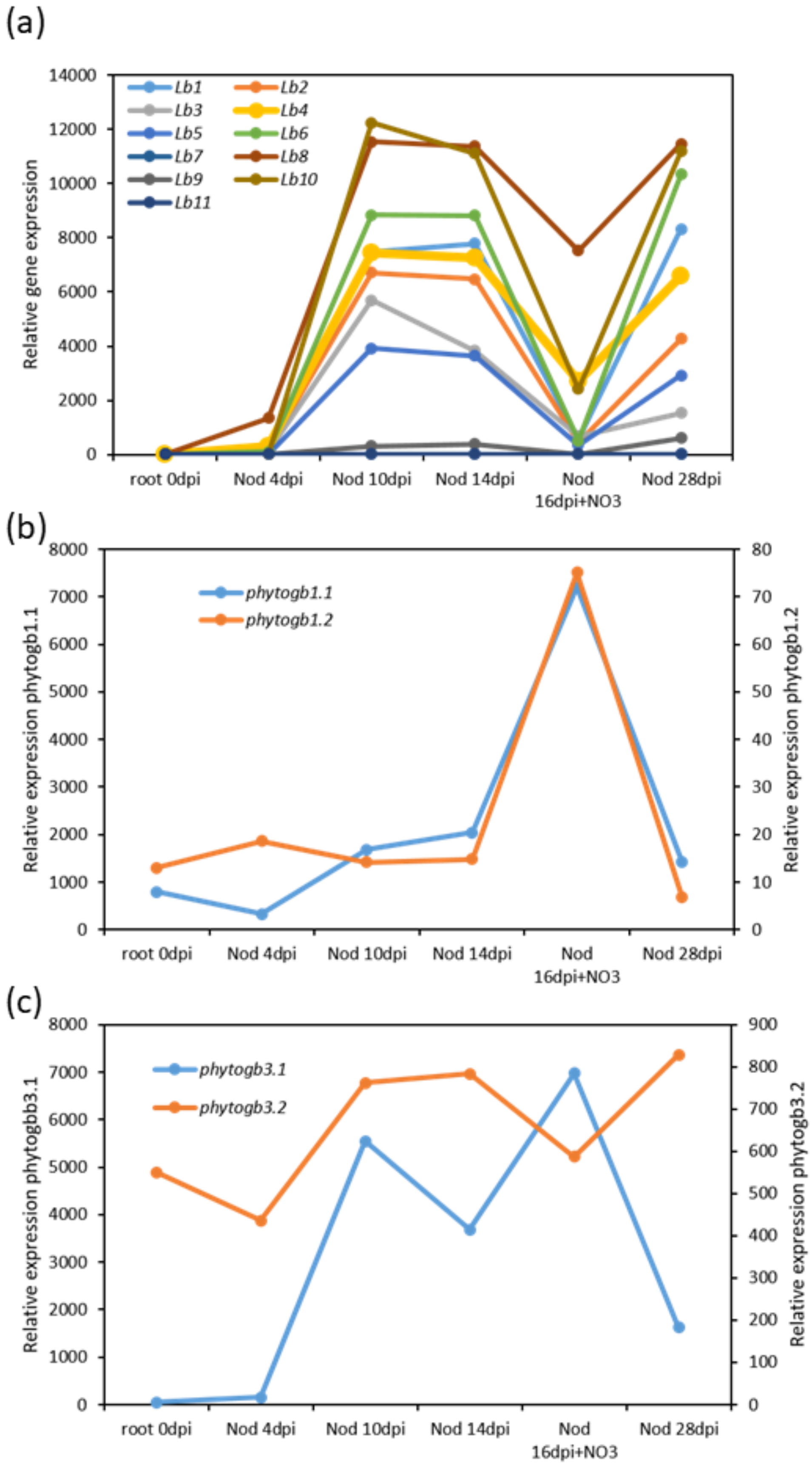

Figure S3: Microarray data for *Medicago truncatula* Phytoglobins. Microarray data were obtained on (https://mtgea.noble.org/v3/).

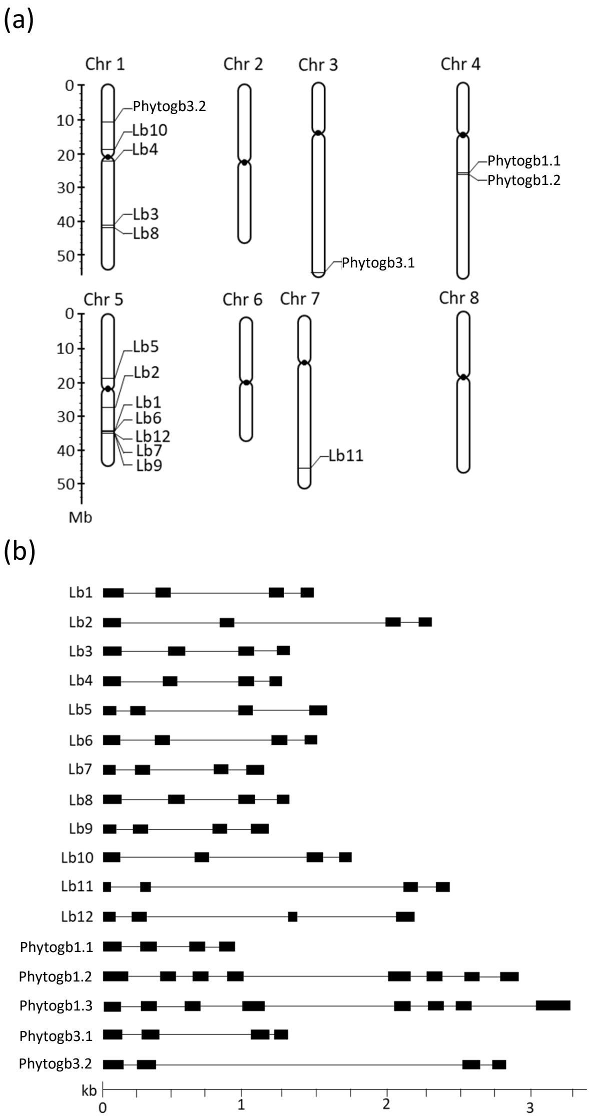

Figure S4: **(a) Chromosomal localisation and (b) exons-introns structure of *Medicago truncatula Phytogb* genes**. No information is available on the chromosomal location of *Phytogb1.3*. Exons are materialized by black box. Mb: megabase; kb: kilobase.

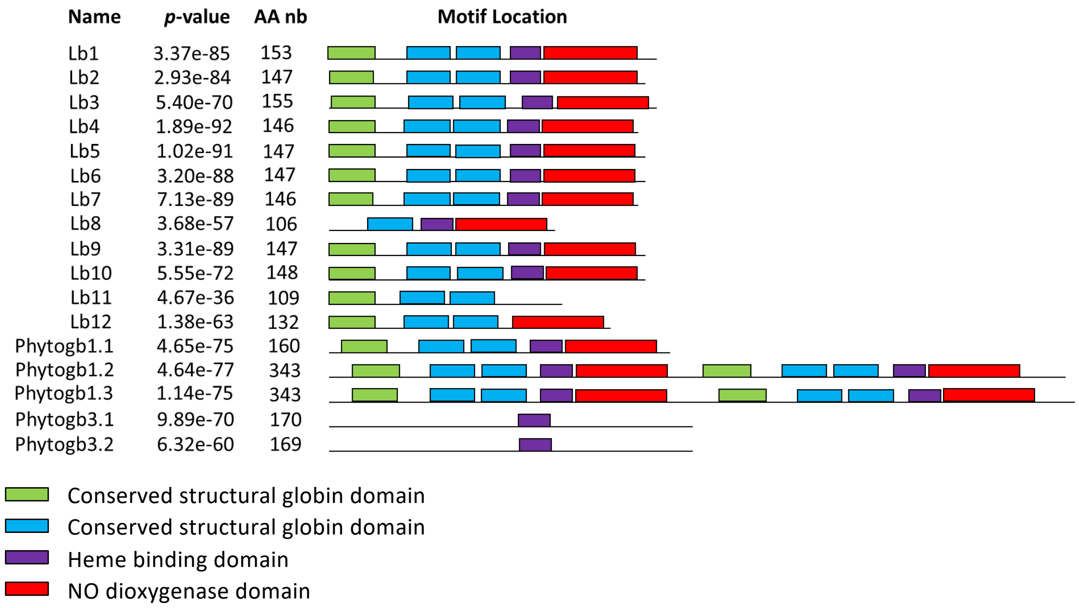

Figure S5: **MEME model of primary sequences of *Medicago truncatula* Phytogb proteins**. Four MEME motifs are identified by the software on the 17 proteins. The location of these motifs represented by color boxes is aligned with the primary sequence of proteins. The p-value of each MEME model of the proteins is indicated as well as the number in amino acids.

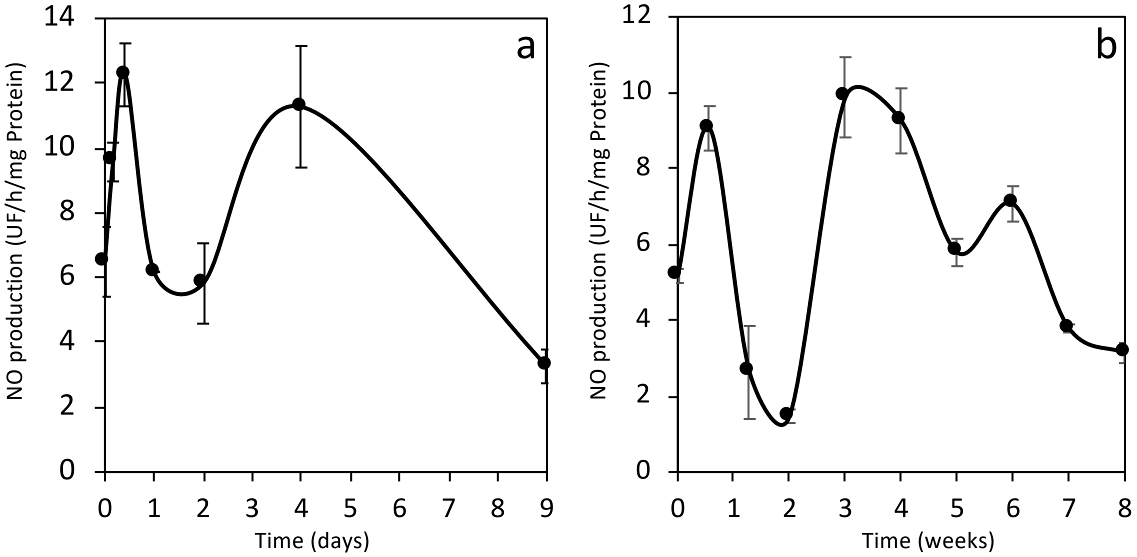

Figure S6: Variations of NO level during the symbiotic process expressed as a function of protein content. Data are drawn from those in Figure 2. (a) Short term kinetic 14 dpi, (b) long-term kinetic 8 wpi. The fluorescence intensity of the NO production was measured using the DAF-2 fluorescent probe. Data are means ± SE (n=3). Each measure was realized in three technical replicates.

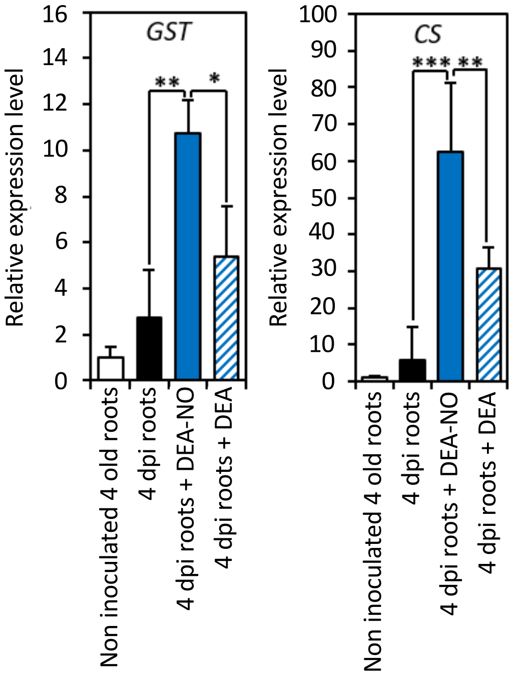

Figure S7: **Defense gene (*GST* and *CS*) expression after 4 days of NO donor treatment.** Plant roots were either inoculated with *S. meliloti* in the presence or absence of 0.5mM DEA/DEA-NO, or not inoculated (Control), and grown for four days before ARN extraction and analysis. Data are means ± SE (n=3). Each measure was realized in three technical replicates. Asterisks indicate statistical differences at P< 0.05 *, P<0.01 **, and P<0.001 ***, according to the Student's *t*-test.

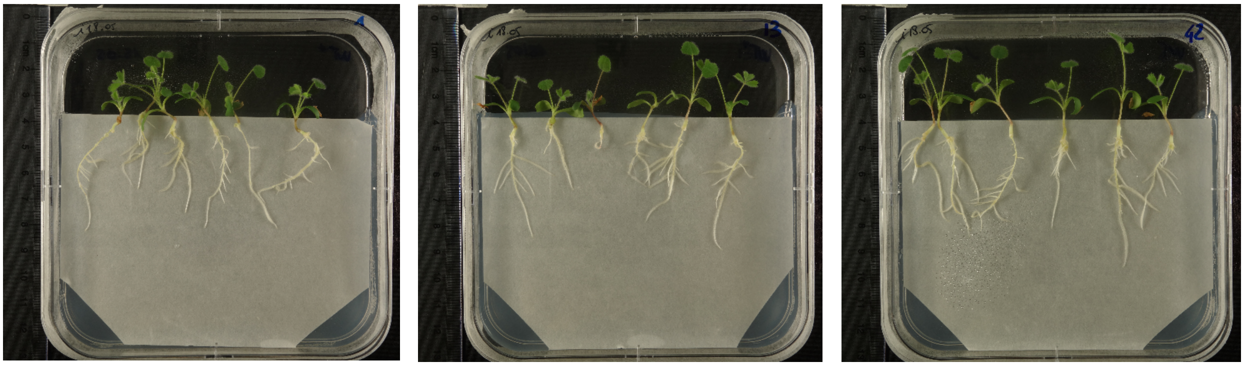

Control *35s::Phytogb1.1* *RNAi::Phytogb1.1*

Figure S8: **Pictures of Control, *35s::Phytogb1.1* and *RNAi::Phytogb1.1* transgenic hairy roots growth on Petri dishes two days after inoculation with *Sinorhizobium meliloti*.**

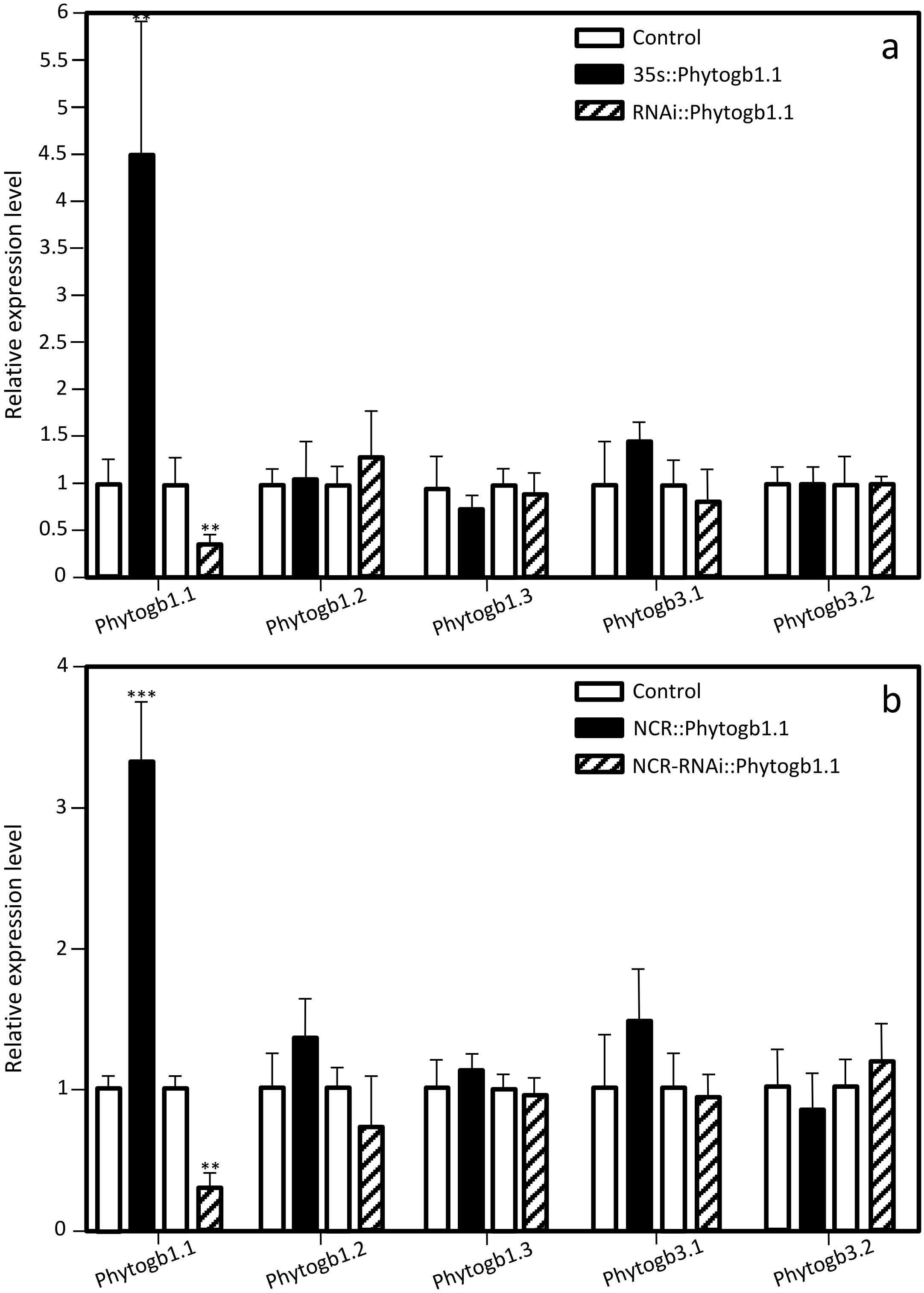

Figure S9: Relative expression level of class 1 and 3 *Phytogb* genes in *Phytogb1.1* transformed roots. Expression analysis of *Phytogb1.1, 1.2, 1.3, 3.1 and 3.2* genes, in control (relative expression level reduced to 1) and transformed plant roots either overexpressing (35s::*Phytogb1.1*) or silencing *Phytogb1.1* (RNAi::*Phytogb1.1*) at 4 dpi. Data are means ± SE (n=3). Each measure was realized in three technical replicates.

Reference

Boscari A, Del Giudice J, Ferrarini A, Venturini L, Zaffini A-L, Delledonne M, Puppo A. **2013**. Expression dynamics of the Medicago truncatula transcriptome during the symbiotic interaction with Sinorhizobium meliloti: which role for nitric oxide? *Plant Physiology* **161**: 425–39.

del Giudice J, Cam Y, Damiani I, Fung-Chat F, Meilhoc E, Bruand C, Brouquisse R, Puppo A, Boscari A. **2011**. Nitric oxide is required for an optimal establishment of the Medicago truncatula-Sinorhizobium meliloti symbiosis. *New Phytologist* **191**: 405–417.

Pierre O, Hopkins J, Combier M, Baldacci F, Engler G, Brouquisse R, Hérouart D, Boncompagni E. **2014**. Involvement of papain and legumain proteinase in the senescence process of Medicago truncatula nodules. *New Phytologist* **202**: 849–863.
